# An Individual-Based Diploid Model Predicts Limited Conditions Under Which Stochastic Gene Expression Becomes Advantageous

**DOI:** 10.3389/fgene.2015.00336

**Published:** 2015-11-24

**Authors:** Tomotaka Matsumoto, Katsuhiko Mineta, Naoki Osada, Hitoshi Araki

**Affiliations:** ^1^Graduate School of Systems Life Sciences, Kyushu UniversityFukuoka, Japan; ^2^Department of Population Genetics, National Institute of GeneticsMishima, Japan; ^3^Computational Bioscience Research Center (CBRC), King Abdullah University of Science and Technology (KAUST)Thuwal, Saudi Arabia; ^4^Department of Genetics, SOKENDAI (The Graduate University for Advanced Studies)Mishima, Japan; ^5^Research Faculty of Agriculture, Hokkaido UniversitySapporo, Japan

**Keywords:** stochastic gene expression, environmental change, viability selection, effective population size, individual-based simulation

## Abstract

Recent studies suggest the existence of a stochasticity in gene expression (SGE) in many organisms, and its non-negligible effect on their phenotype and fitness. To date, however, how SGE affects the key parameters of population genetics are not well understood. SGE can increase the phenotypic variation and act as a load for individuals, if they are at the adaptive optimum in a stable environment. On the other hand, part of the phenotypic variation caused by SGE might become advantageous if individuals at the adaptive optimum become genetically less-adaptive, for example due to an environmental change. Furthermore, SGE of unimportant genes might have little or no fitness consequences. Thus, SGE can be advantageous, disadvantageous, or selectively neutral depending on its context. In addition, there might be a genetic basis that regulates magnitude of SGE, which is often referred to as “modifier genes,” but little is known about the conditions under which such an SGE-modifier gene evolves. In the present study, we conducted individual-based computer simulations to examine these conditions in a diploid model. In the simulations, we considered a single locus that determines organismal fitness for simplicity, and that SGE on the locus creates fitness variation in a stochastic manner. We also considered another locus that modifies the magnitude of SGE. Our results suggested that SGE was always deleterious in stable environments and increased the fixation probability of deleterious mutations in this model. Even under frequently changing environmental conditions, only very strong natural selection made SGE adaptive. These results suggest that the evolution of SGE-modifier genes requires strict balance among the strength of natural selection, magnitude of SGE, and frequency of environmental changes. However, the degree of dominance affected the condition under which SGE becomes advantageous, indicating a better opportunity for the evolution of SGE in different genetic models.

## Introduction

The level of gene expression is one of the important factors which affects an individual's fitness. Many studies have shown that organisms have changed the expression level of certain genes and adapted to new habitats (Maury et al., 2008; Stern and Orgogozo, 2008; Fraser, 2013). Intuitively, natural selection has shaped the levels and patterns of gene expression, and strict control of the gene expression levels by genotype must be highly advantageous. However, recent studies suggested that for many organisms, a stochasticity in gene expression (SGE) varies phenotypes even across individuals in isogenic populations (Shahrezaei and Swain, 2008a). For example, Acar et al. (2008) engineered a yeast strain that expresses multiple phenotypes due to SGE. Similary, Tsuru et al. (2011) engineered *E. coli* strain and found that a SGE made a phenotype which can survive in histidine disturbed condition. Even in multicellular organisms, SGE has been reported to be beneficial in cell-fate determination (Colman-Lerner et al., 2005; Kaern et al., 2005; Losick and Desplan, 2008; Gomes et al., 2011) and cell growth (Fraser et al., 2004; Newman et al., 2006; Batada and Hurst, 2007; Lehner, 2008).

Throughout this study, we refer SGE as stochasticity in gene expression that causes stochastic variation of a hypothetical phenotype which is defined for each individual and subject to natural selection (See Mineta et al., 2015 for more details). Note that the phenotypic variation of interest in this study is among individuals, and that we do not specifically study variations of gene expression along the time (such as circadian rhythm- or developmental stage-dependent gene expression) or among organs. The major consequence of SGE is a provision of stochastic phenotypic variation and differences in fitness among individuals even in an isogenic population. The importance of SGE has been predicted in several theoretical studies as a “bet-hedging” strategy, for instance in microbes in fluctuating environments (Kussell and Leibler, 2005). They compared the growth rates of two populations, with and without stochastic switching in phenotype, and found that the stochastic switching can be favored under infrequent environmental fluctuation. Acar et al. (2008) also theoretically compared the growth rates of populations assuming two phenotypes with the stochastic phenotypic switch. They suggested that fast phenotypic switching becomes advantageous in quickly fluctuating environments. In addition, Salathe et al. (2009) incorporated asymmetric fitness landscape, in which the cost of being maladaptive is different in different environments. They found that under the asymmetric fitness landscape, the evolution of a stochastic phenotypic switching became difficult. These theoretical studies were excellent examples of how SGE can potentially be associated with evolution in the biological context, although they used rather complex and sometimes very specific models of SGE. Thus, the general importance of SGE in molecular evolution is still largely an open question.

In the present study, we performed individual-based computer simulations to quantitatively evaluate the condition in which enhanced SGE can be advantageous in fluctuating environments. Previous studies suggested that SGE can be caused by both extrinsic factors like transcription factor activity, and intrinsic factors like mRNA decay ratio (Elowitz et al., 2002; Raser and O'shea, 2005). Therefore, SGE itself is non-genetic, but regulatory systems that enhance or reduce the magnitude of SGE could be controlled by genetic mechanisms (Mineta et al., 2015). If the effect of SGE on fitness is environment-dependent, and if the magnitude of SGE is regulated by a genetic component (“SGE modifier gene” in Mineta et al., 2015), such a genetic component might evolve and enhance the magnitude of SGE under certain conditions. The production and degradation rates of protein and mRNA critically are known to affect the magnitude of SGE (Shahrezaei and Swain, 2008b; Li and Xie, 2011; Grima et al., 2012), implying that the genes regulating the production-degradation pathway might be SGE modifier gene candidates.

**Figure 1:**
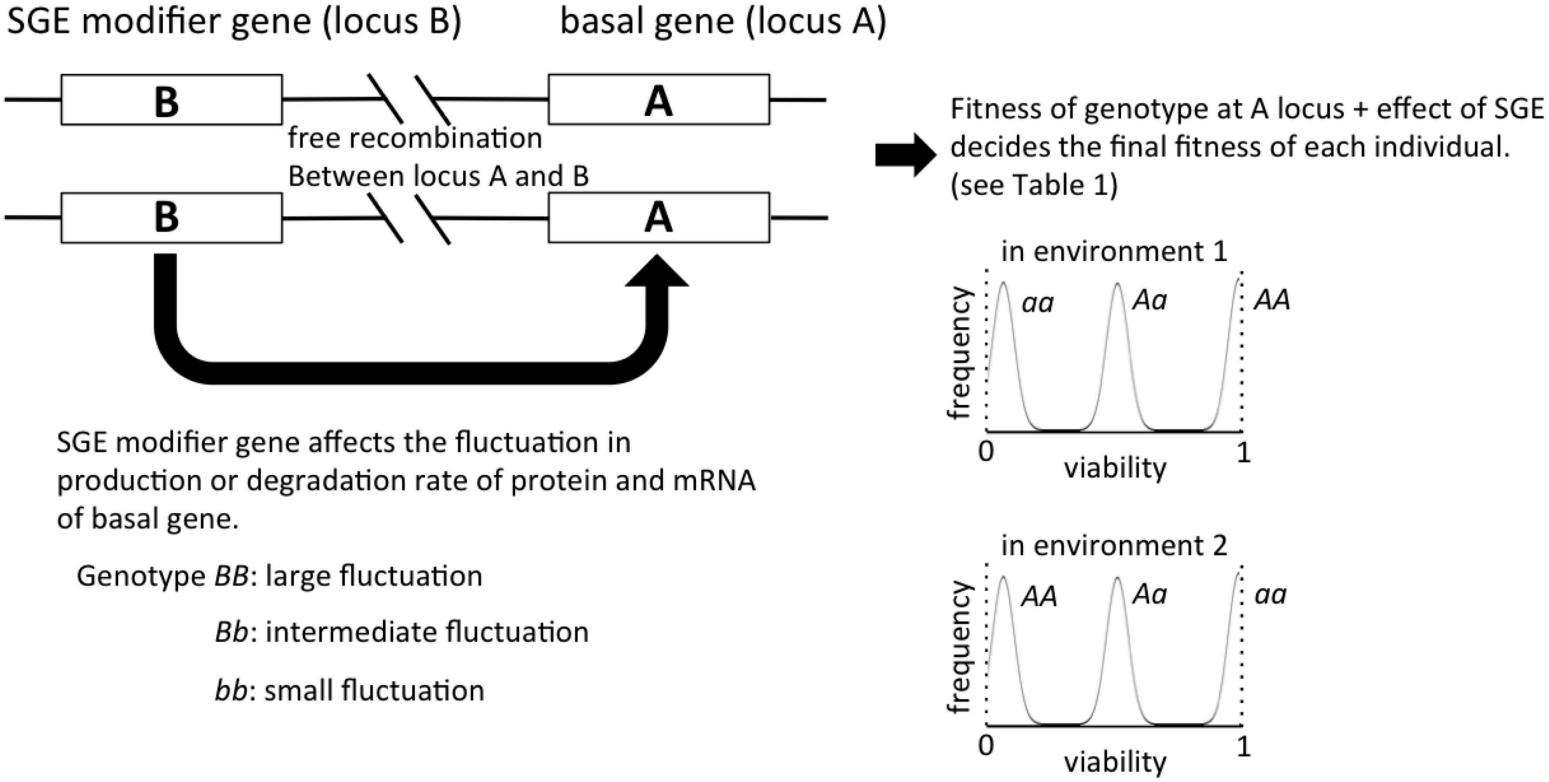
**The outline of the individual-based model in this study**. The viability of each individual is primarily determined by the genotype of locus A, and the magnitude of SGE, which causes fitness deviation, is regulated by locus B. In our model of viability selection, viability 0 and 1 are assumed to be absorbing boundaries.

In the current study, we considered two loci: first we constructed a simple population genetic model assuming that individual phenotype was basically determined by one locus. The allele set of this locus provides the basal genotype, which primarily determines the individual phenotype, which in turn is subject to stochastic deviation due to SGE given by a Gaussian function (Figure 1). Second, we considered the other locus that regulates the magnitude of SGE (i.e., SGE modifier gene). The fitness of organisms was then determined by their basal genotypes and stochastic variation, whereas the level of fitness variation was determined by the genotype at the modifier locus in our model. Recently developed models on SGE tend to incorporate relationships among several processes, which take into consideration noises in transcription, translation, mRNA and protein decay (Draghi and Whitlock, 2015; Wolf et al., 2015). Our model was constructed to be rather simple because we wanted to address the questions whether and when SGE and its modifier gene can evolve more in general, although we acknowledge the fact that part of our assumptions might be too simple to explain specific empirical data.

Conducting the individual-based simulations above, we evaluated the conditions in which the frequency of SGE-enhancing alleles can increase in the population. Although we intended to evaluate the predictions made by Mineta et al. (2015) above, there is an important difference between the assumptions behind this study and those made by Mineta et al. (2015). In Mineta et al. (2015), the symmetric fluctuation of fitness by SGE was assumed. Therefore, the SGE modifier gene was basically assumed to evolve selectively neutrally under stable environmental conditions in the previous study. However, if an individual has a highly advantageous basal genotype in the environment, SGE which fluctuates the phenotype around the optimal may cause more disadvantageous effects than advantageous effects. Similarly, SGE may cause more advantageous effects when an individual's basal genotype is highly disadvantageous. To address this issue, we assumed viability selection with 0–1 fitness boundary in the present study. If the phenotype is affected by strong viability selection rather than fecundity selection, a certain level of SGE may decrease the viability of individuals to 0. In such cases, viability = 0 acts as the absorbing boundary so that even if the effect of SGE on the phenotype is symmetric, the average effect of SGE on viability can be advantageous. Similarly, SGE occurring in the optimal basal genotype can be disadvantageous because viability = 1 is also the absorbing boundary (for another means of making the evolutionary effect of SGE asymmetric, see Zhang et al., 2009). Previously, many studies have reported that there are examples showing that the expression level of a single or a few major genes which relate to stress or disease tolerance can dramatically change individuals' viability (Hou et al., 2009; Morran et al., 2011; Shi et al., 2012; Yang et al., 2012; Ravaux et al., 2013). Therefore, SGE acting on these genes would potentially have a large effect on viability. In such cases, the evolution of the SGE modifier gene might be influenced by the asymmetric SGE around the viability boundary.

## Model

### Individuals

Individuals are assumed to be diploid and monoecious, and two loci (locus A and B) were considered in our model (Figure 1). Locus A regulates the trait directly affected by natural selection, and locus B regulates the magnitude of SGE. For simplicity, we assumed that each locus has two alleles (allele *A* and *a* for locus A, allele *B* and *b* for locus B), and the degree of dominance was manipulated. The fitness of each individual was determined by the genotype and the effect of SGE (see Viability Selection section). In this model, we assumed free recombination between loci, and we did not consider new mutation. The linkage disequilibrium might alter the fate of SGE evolution, but it is beyond the scope of the current study. The code for our simulations can be provided by T.M. upon request.

### Environmental change

We consider the fluctuating environment assuming that two environments, environment 1 and environment 2, switch every *T* generation. In the different environment, each individual is affected by different viability selection.

### Viability selection

In environment 1, the viability of individuals with three genotypes at locus A, *AA, Aa*, and *aa*, is defined as 1, 1 − *h*_1_*S*, and 1 − *S*, respectively, where *h*_1_ is the degree of dominance (0 ≤ *h*_1_ ≤ 1) and *S* is the selection coefficient. In contrast, the viability of individuals with the genotypes *AA, Aa*, and *aa* becomes 1 − *S*, 1 − *h*_2_*S*, and 1, respectively (0 ≤ *h*_2_ ≤ 1), in environment 2. Therefore, allele *A* becomes adaptive in environment 1, and allele *a* becomes adaptive in environment 2. In addition, the viability is expected to vary among individuals due to SGE (Wang and Zhang, 2011). A normally distributed random variable *q* with mean 0 and standard deviation σ is considered, which represents the effect of SGE on viability of individuals in addition to the effect of locus A. Later, we consider the effect of locus B, which influences the value of σ. Namely, each genotype at locus B (*BB, Bb*, and *bb*) defines a different level of σ (σ_*BB*_, σ_*Bb*_, and σ_*bb*_), and we assumed σ_*BB*_ ≥ σ_*Bb*_ = *h*_*b*_σ_*BB*_ ≥ σ_*bb*_ = 0 (0 ≤ *h*_*b*_ ≤ 1). Thus, in environment 1, the viability of individuals with genotypes *AA, Aa*, and *aa* can be described as 1 + *q*, 1 − *hS* + *q*, and 1 − *S* + *q*, respectively, and locus B affects the range of *q* (Table 1). Note that in this study, we assume that viability 0 and 1 are the absorbing boundary, and viability lower than 0 and higher than 1 are considered as viability 0 and 1, respectively. Because locus B regulates the magnitude of SGE, we call this locus SGE modifier gene in the following sections. σ_*bb*_ = 0 (no SGE due to the genotype *bb*) would be an unrealistic assumption in the real world, but it was set as such in order to illuminate the effect of SGE modifier gene on fitness.

**Table 1:** **Viability of three genotypes *AA, Aa*, and *aa* in two environments**.

	***AA***	***Aa***	***aa***
Environment 1	1 *+ q*	1 −*h*_1_*S* + *q*	1 −*S* + *q*
Environment 2	1 −*S* + *q*	1 −*h*_2_*S* + *q*	1 *+ q*

### Mating

After the viability selection, surviving individuals mate to create the offspring population. In this model, we assume random mating with constant population size *N* and discrete generations. Therefore, mating pairs are randomly chosen to allow a replication from the surviving individuals and make the offspring until the population size reaches *N*. These offspring become the parental population in the next generation. We assume that each mating pair can make only one offspring per mating event.

## Results

### Increased fixation probability of deleterious mutations due to SGE

Mineta et al. (2015) predicted that SGE can reduce effective population size and thus, fixation probability of slightly deleterious mutations might be high under SGE. First, we tested this prediction in our model, assuming fixation of allele *B* at locus B (i.e., σ = σ_*BB*_). We set *N* = 1000, *h*_1_ = 0.5 (no dominance) and *T* = infinite (stable environment 1), and considered the initial condition that the frequency of advantageous allele *A* is (2*N* − 1)/2*N* and deleterious allele *a* is 1/2*N* at locus A. Starting from this condition, we simulated how many times the deleterious allele could fix in the population under variable *S* and σ. We considered four *S*-values representing different levels of natural selection; *S* = 0.00025, 0.0005, 0.00125, 0.0025, and 0.005 (2*NS* = 0.5, 1.0, 2.5, 5.0, and 10.0, respectively). We also examined five σ_*BB*_ values; σ_*BB*_ = 0 (without SGE), 0.001, 0.01, 0.1, 1.0. The simulation was replicated 50,000 times in each parameter set.

**Figure 2:**
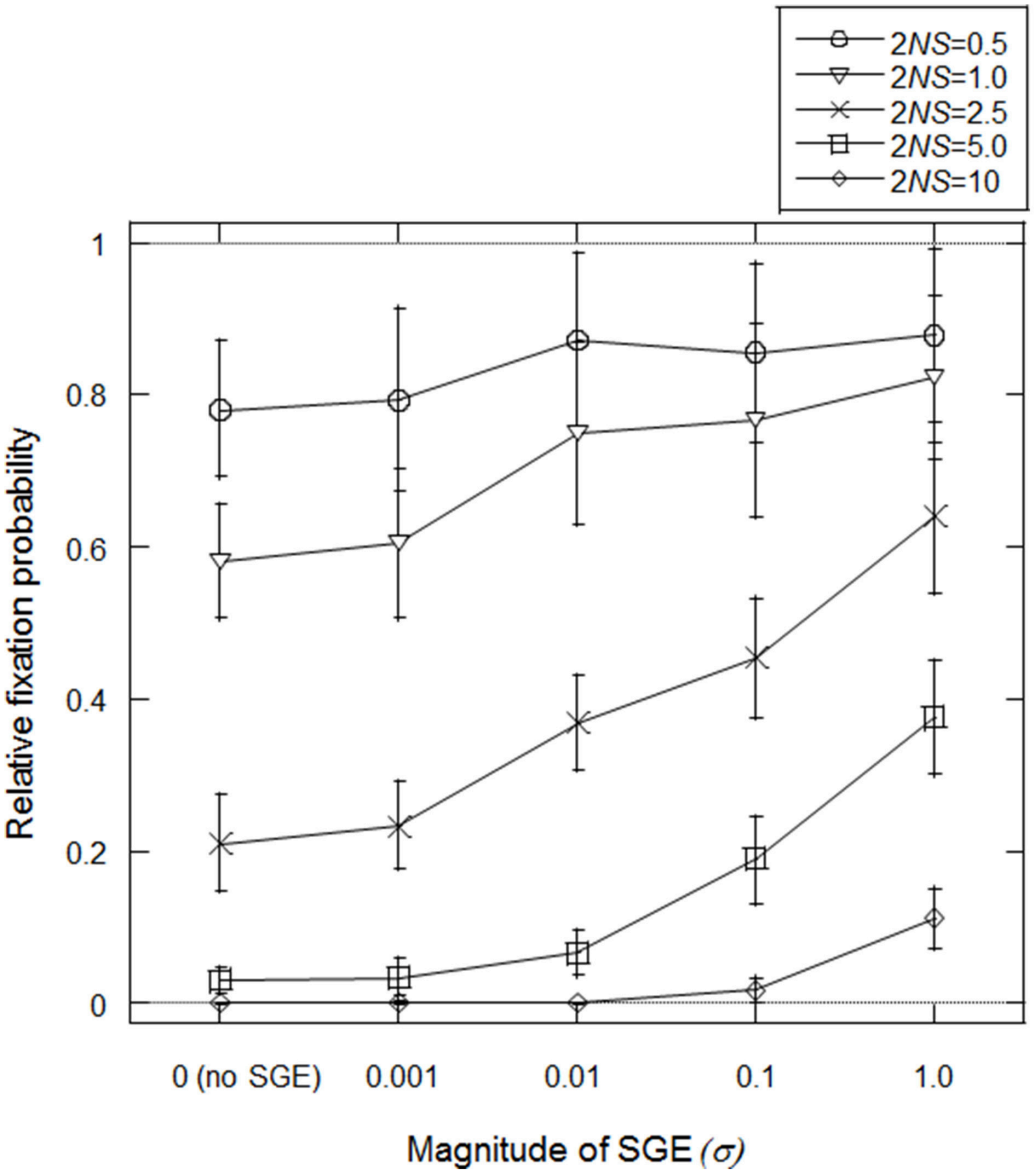
**Average fixation probability of deleterious allele (*a*) relative to neutral expectation (1/2*N*)**. Population size (*N*) was fixed to be 1000 and different natural selection strengths (2*NS*) were examined by changing selection coefficient (*S*) under different magnitudes of SGE (given by the standard deviation of SGE fitness effect, σ). Five different selection coefficients, S = 0.00025, 0.0005, 0.00125, 0.0025, and 0.005 (2*NS* = 0.5, 1.0, 2.5, 5.0, and 10.0, respectively), and five different magnitudes of SGE, σ = 0 (without SGE), 0.001, 0.01, 0.1, 1.0 were considered. Viability of each genotype was given as *AA* = 1 + *q, Aa* = 1 − 0.5*S* + *q*, and *aa* = 1 − *S* + *q*, where *q* represents SGE fitness effect by choosing a random value from normal distribution with mean = 0 and standard deviation = σ. Initial frequency of *a* was 1/2*N*. Fixation probability was calculated among 50,000 replicates in each parameter set assuming constant population size, random mating, and no mutation, and this process was replicated 100 times to calculate the average and standard deviation of the fixation probability.

In Figure 2, fixation probabilities, relative to the expected fixation probability under strict neutrality (= 1/2*N*), were measured with/without SGE and with different strengths of natural selection. A relative increase in the fixation probability was observed with SGE, as opposed to without, particularly when σ and 2*NS* were large, suggesting that the positive effect of SGE on the fixation probability of deleterious mutation was larger when the allele exhibited a more deleterious effect. However, even under very strong effects of SGE (σ_*BB*_ = 1.0), the fixation probability of the deleterious alleles never became higher than in the case of strict neutrality (i.e., relative fixation probability of 1.0 in Figure 2). As shown in Mineta et al. (2015), SGE gradually decreases the effective population size as the number of generations increase. Thus, although SGE greatly increased the fixation probability of the deleterious mutation compared to the cases without SGE, SGE is most likely subject to purifying selection in these conditions.

### Evolutionary fate of SGE modifier gene under stable and fluctuating environment

**Figure 3:**
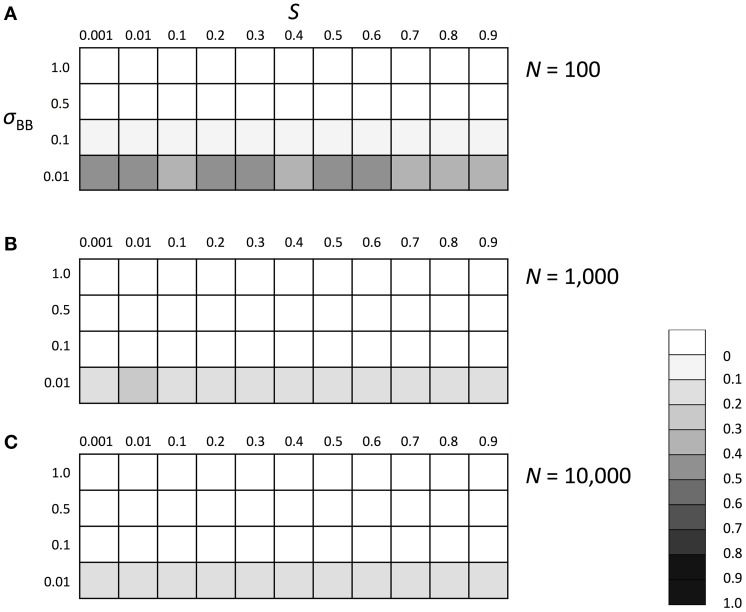
**The average frequency of SGE enhancing allele *B* under stable environment 1**. Viability of each genotype in this environment was 1 + *q*, 1 − *h*_1_*S* + *q*, and 1 − *S* + *q* for *AA, Aa*, and *aa*, respectively. The magnitude of SGE was regulated by locus B as σ_*BB*_ ≥ σ_*Bb*_ = *h*_*b*_σ_*BB*_ ≥ σ_*bb*_ = 0 (0 ≤ *h*_*b*_ ≤ 1). As the initial condition, Hardy-Weinberg equilibrium was assumed for locus A and B. Parameters *h*_1_ and *h*_*b*_ were set to 0.5, and *T* was set to infinite. Different panels show the result in different population size: **(A)** *N* = 100, **(B)** *N* = 1000, and **(C)** *N* = 10, 000. The simulation was replicated 1000 times for each parameter set and gray scale shows the average frequency of allele *B* after 1000 generations. In the simulation, constant population size, random mating, free recombination, and no mutation were assumed.

We subsequently investigated the evolutionary fate of the genetic regulation on SGE. Initially, we examined which of the enhanced or suppressed SGE was advantageous in a stable environment (environment 1). We considered Hardy-Weinberg equilibrium for both loci (A and B) with allele frequencies of 0.5 as the initial condition. Three population sizes, *N* = 100, 1000, and 10,000, were examined to evaluate the effect of the population size and selection coefficient separately. For other parameters, we set *h*_1_ = *h*_*b*_ = 0.5 and *T* = infinite. By changing the selection coefficient of locus A (*S*) and the magnitude of SGE (σ_*BB*_), we examined whether the frequency of allele *B*, which enhances the magnitude of SGE, could increase in the population or not. The simulation was replicated 1000 times in each parameter set; if the average frequency of allele *B* was larger than 0.5 after 1000 generations, we considered that the enhanced SGE was advantageous. Although 1000 generations is too short to get the equilibrium state in large population size, our strategy to observe the change of the average frequency of allele *B* from the initial frequency 0.5 allows us to evaluate whether the enhanced SGE is advantageous or not, even within a short generation. As the result, in the stable environment, the allele frequency of *B* was substantially lower than 0.5 in all conditions, indicating that enhanced SGE was disadvantageous compared with suppressed SGE and therefore, decreased its frequency after long generations (Figure 3). In *N* = 100, the effect of genetic drift became large, which caused the higher frequency of *B* than that in *N* = 1000 and 10,000 (Figure 3A). In Supplementary Figures 1, 2, we show the average frequency of allele *A* and *B* in each generation with *N* = 1000 and *S* = 0, 0.5, and 0.9 as the representatives. If *S* was larger than 0, allele *A* became advantageous in environment 1 and increased in frequency and finally fixed (Supplementary Figure 1, if *S* = 0, both of allele *A* and *a* had the same fitness and thus, the average frequency of *A* was about 0.5). Allele *B* frequency never increased in the population (Supplementary Figure 2), suggesting that SGE decreased the average fitness in the population by causing the deviation from the optimal phenotype, and acted as a load as predicted by Mineta et al. (2015). The disadvantageous effect of allele *B* increased as *S* and σ_*BB*_ became large.

**Figure 4:**
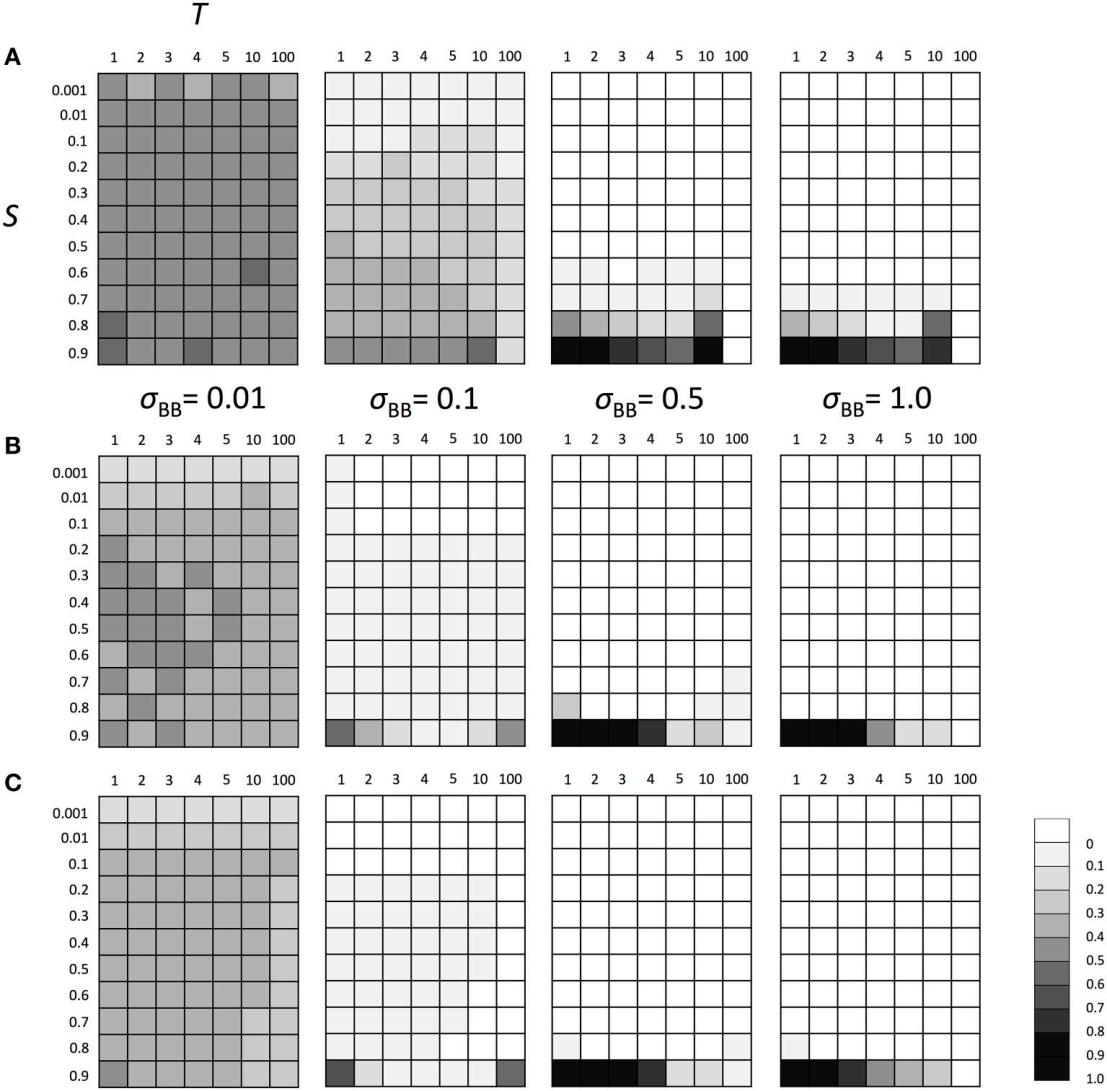
**The average frequency of SGE enhancing allele *B* under fluctuating environments**. Viability of each genotype 1 + *q*, 1 − *h*_1_*S* + *q*, and 1 − *S* + *q* in environment 1, and 1 − *S* + *q*, 1 − *h*_2_*S* + *q*, and 1 + *q* in environment 2 for *AA, Aa*, and *aa*, respectively. Two environments switched every *T* generation. The magnitude of SGE was regulated by locus B as σ_*BB*_ ≥ σ_*Bb*_ = *h*_*b*_σ_*BB*_ ≥ σ_*bb*_ = 0 (0 ≤ *h*_*b*_ ≤ 1). As the initial condition, Hardy-Weinberg equilibrium was assumed for locus A and B. Parameters *N* was set to 1000 and *h*_1_, *h*_2,_ and *h*_*b*_ were set to 0.5. In this simulation, four σ_*BB*_ values, σ_*BB*_ = 1.0, σ_*BB*_ = 0.5, σ_*BB*_ = 0.1, and σ_*BB*_ = 0.01 were considered. Different panels show the result in different population size: **(A)** *N* = 100, **(B)** *N* = 1000, and **(C)** *N* = 10,000. The simulation was replicated 1000 times for each parameter set, and gray scale shows the average frequency of allele *B* after 1000 generations. In the simulation, constant population size, random mating, free recombination, and no mutation were assumed.

Next, we examined the evolutionary fate of the SGE modifier gene in fluctuating environments. Similarly to the above scenarios, we considered Hardy-Weinberg equilibrium for all loci A and B as the initial condition and three population sizes (*N* = 100, *N* = 1000, and *N* = 10,000). We also set *h*_1_ = *h*_2_ = *h*_*b*_ = 0.5. The switch between two different environments 1 and 2 was caused every *T* generations (initial environment was 1). As explained in the Model section, individuals are affected by different selection in different environments (Table 1). Thus, as shown in Supplementary Figure 3, polymorphism at locus A was maintained during the simulation and the population could adapt to the current environment using this polymorphism. However, just after the environmental change, most individuals would have low fitness and SGE could be adaptive. As shown in Figure 4, the enhanced SGE could be advantageous only in very restricted conditions; when the natural selection in each environment was strong, environment switched frequently and the magnitude of SGE was large (e.g., fixation of the allele *B* in the population in *N* = 1000, *S* = 0.9, *T* = 1 and large σ_*BB*_; Figure 4B). If the magnitude of SGE was small, allele *B* could be maintained in wide parameter range, but the frequency was smaller than 0.5 in *N* = 1000 and 10,000, suggesting that enhanced SGE was not advantageous overall (Figures 4B,C). We confirmed that the allele *B* was always lost from the population after 10,000 generations in these cases (data not shown). Exceptionally, in *N* = 100, the combination of *T* = 10, large *S* and σ_*BB*_ caused the increase of allele *B* frequency (Figure 4A). This is presumably because polymorphism at locus A was lost in these cases. Without polymorphism at locus A, adaptation of the locus directly affected by natural selection is impossible, and the advantageous effect of SGE might increase. Fixation of the allele at locus A would occur more frequently in *T* = 100. In this case, however, long intervals between environmental switches also caused the loss of allele *B* before the first environmental switch. This is presumably why allele *B* frequency did not increase in the population (Figure 4A). Only when population size was large (*N* = 1000, 10,000) and the magnitude of SGE was small (σ_*BB*_ = 0.1), allele *B* could be maintained until the first environmental switch. In such cases, allele *B* frequency could increase in the population (Figures 4B,C). Thus, high frequencies of allele *B* in *T* = 10 and 100 was a consequence of the loss of polymorphism at locus A. As shown in the next paragraph, without polymorphism at locus A, allele *B* frequency increased even with large *T*, which is consistent with the result shown in Figure 4A. With *N* = 100 and σ_*BB*_ = 0.01, the frequency of allele *B* was around 0.5 suggesting that the evolution of allele *B* may occur almost neutrally in these cases. In Supplementary Figure 4, we show the frequency change of allele *B* in each generation when *N* = 1000, *S* = 0.8 or 0.9 and *T* = 1 or 10. As consistent with the result in Figure 4B, the allele *B* could be advantageous and increase its frequency only under extremely strong selection (*S* = 0.9) and frequent environment switch (*T* = 1; Supplementary Figure 4D).

**Figure 5:**
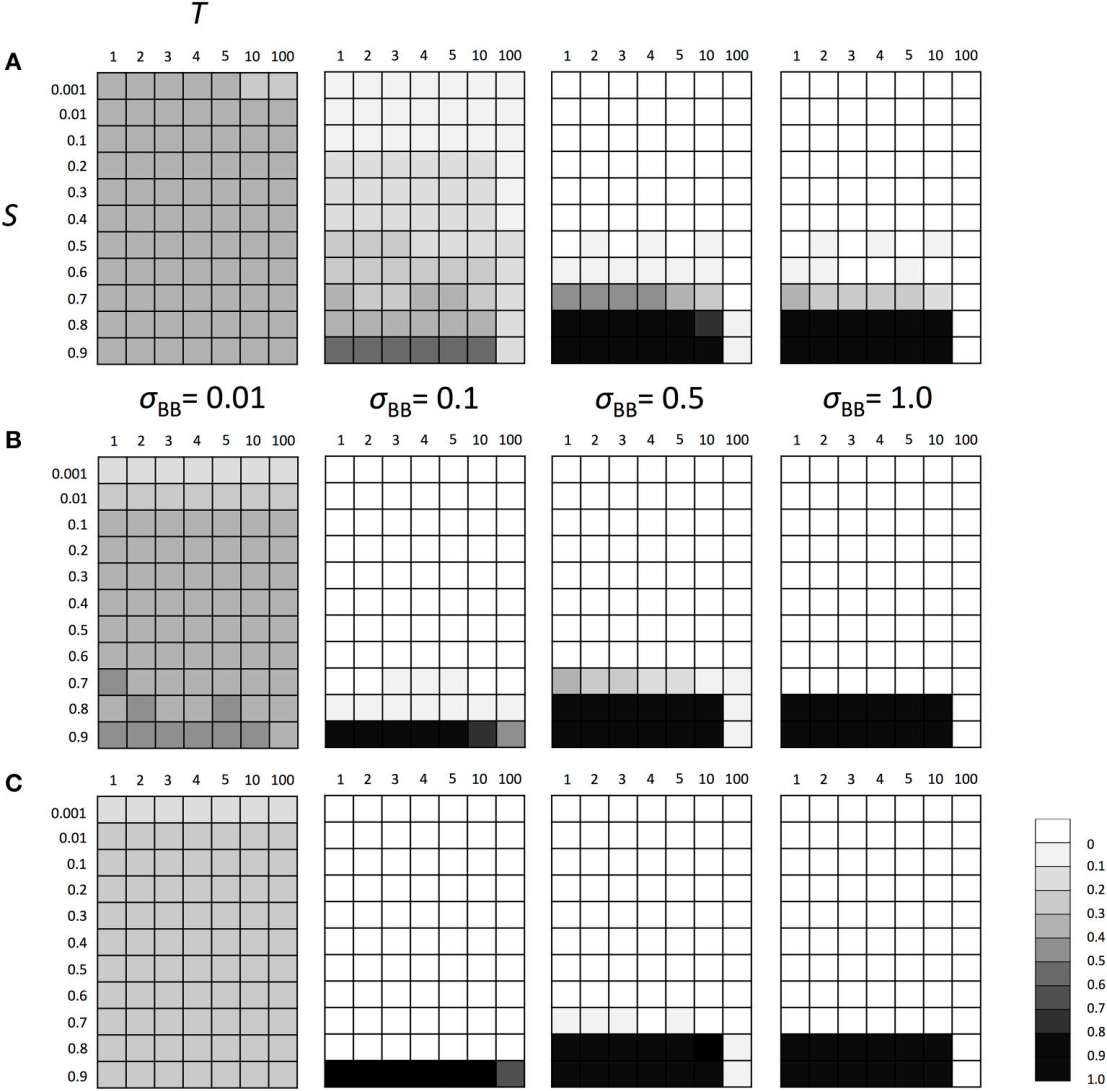
**The average frequency of SGE enhancing allele *B* under fluctuating environments assuming fixed allele *A***. Viability of genotype *AA* was 1+ *q* in environment 1 and 1 − *S* + *q* in environment 2. Two environments switched every *T* generation. The magnitude of SGE was regulated by locus *B* as σ_*BB*_ ≥ σ_*Bb*_ = *h*_b_^*^ σ_*BB*_ ≥ σ_*bb*_ = 0 (0 ≤ *h*_*b*_ ≤ 1). In this simulation, four σ_*BB*_ values, σ_*BB*_ = 1.0, σ_*BB*_ = 0.5, σ_*BB*_ = 0.1, and σ_*BB*_ = 0.01 were considered. As the initial condition, Hardy-Weinberg equilibrium was assumed for locus B and allele *A* was assumed to be fixed at locus A. Parameter *h*_*b*_ was set to 0.5. Different panels show the result in different population size: **(A)** *N* = 100, **(B)** *N* = 1000, and **(C)** *N* = 10, 000. The simulation was replicated 1000 times for each parameter set and gray scale shows the average frequency of allele *B* after 1000 generations. In the simulation, constant population size, random mating, free recombination, and no mutation were assumed.

Above results suggest that the polymorphism at locus A made the adaptation of the locus directly affected by natural selection possible and decreased the advantageous effect of SGE. Thus, if the population did not have the polymorphism at locus A, the importance of SGE might increase in a fluctuating environment. To examine this hypothesis, we conducted the simulation assuming fixation of allele *A* at locus A. We considered the same parameters as we considered above, except for locus A. As we expected, the enhanced SGE could be more advantageous in a wider parameter range under the allele *A* fixation scenario than under the polymorphic scenario (Figure 5) even in *T* = 10. However, the strong selection was still required and the enhanced SGE could not be advantageous in *S* ≤ 0.7. Also in *T* = 100, enhanced SGE could not be advantageous except in σ_*BB*_ = 0.1. As explained above, this is because allele *B* was lost before the first environmental switch in most of the cases. If we assumed initial fixation of allele *a* at locus A, enhanced SGE became advantageous in the initial environment, and allele *B* frequency could increase in the population in a wider parameter range. In Supplementary Figure 5, we show the frequency change of allele *B* in each generation when *N* = 1000, *S* = 0.8 or 0.9 and *T* = 10.

### The effect of the degree of dominance

In the simulations above, we assumed additive effect for both locus A and B. Next, we examined the effect of the degree of dominance for the evolutionary fate of the SGE modifier gene. We considered three values for *h*_1_, *h*_2_, and *h*_*b*_ (0, 0.5, and 1) and simulated the frequency of allele *B* assuming initial Hardy-Weinberg equilibrium at both loci with fluctuating environments. We used *N* = 1000 to examine the effect of the degree of dominance because the above results suggested that the parameter range for allele *B* to be advantageous was not affected by population size except in circumstances where the polymorphism at locus A was no longer present at *N* = 100 (Figure 4A). When *h*_1_ or *h*_2_ = 0, the enhanced SGE could not become advantageous even in extremely strong selection (*S* = 0.9) and frequent environment switch (*T* = 1; Supplementary Figures 6A,B). In these cases, genotype *Aa* can have high fitness on average, and polymorphism at locus A can be maintained for relatively longer generations even when the natural selection is strong (small Δ*q* in Charlesworth and Charlesworth, 2009, p. 55). Therefore, the importance of SGE just after the environmental switch decreased, and it became difficult for the enhanced SGE to be advantageous. On the other hand, high *h*_1_ or *h*_2_ decreased the fitness of heterozygotes and thus, increased the advantage of SGE just after the environmental switch. If we set *h*_1_ or *h*_2_ = 1, the enhanced SGE could be advantageous in a wider parameter range than in *h*_1_ = *h*_2_ = *h*_*b*_ = 0.5 (Supplementary Figures 6C,D). In these cases, allele *B* almost always fixed in *S* = 0.8, *T* = 10, but did not in *S* = 0.7 and *T* = 100.

In contrast to *h*_1_ and *h*_2_, *h*_*b*_ did not have a large effect on the parameter range in which the enhanced SGE could be advantageous (average frequency of allele *B* > 0.5, Supplementary Figure 7). However, small *h*_*b*_ decreased the disadvantageous effect of SGE in heterozygotes and allowed the maintenance of allele *B* in the population (Supplementary Figure 7A). Especially in *T* = 100, fixation at locus A would occur frequently. Thus, if allele *B* was maintained until the first environmental switch, this allele could have a large chance to fix in the population. As a result, average frequency of allele *B* became higher than in shorter *T* cases (Supplementary Figure 7A).

### Evolutionary fate of SGE modifier gene under symmetric effect of SGE on fitness

**Figure 6:**
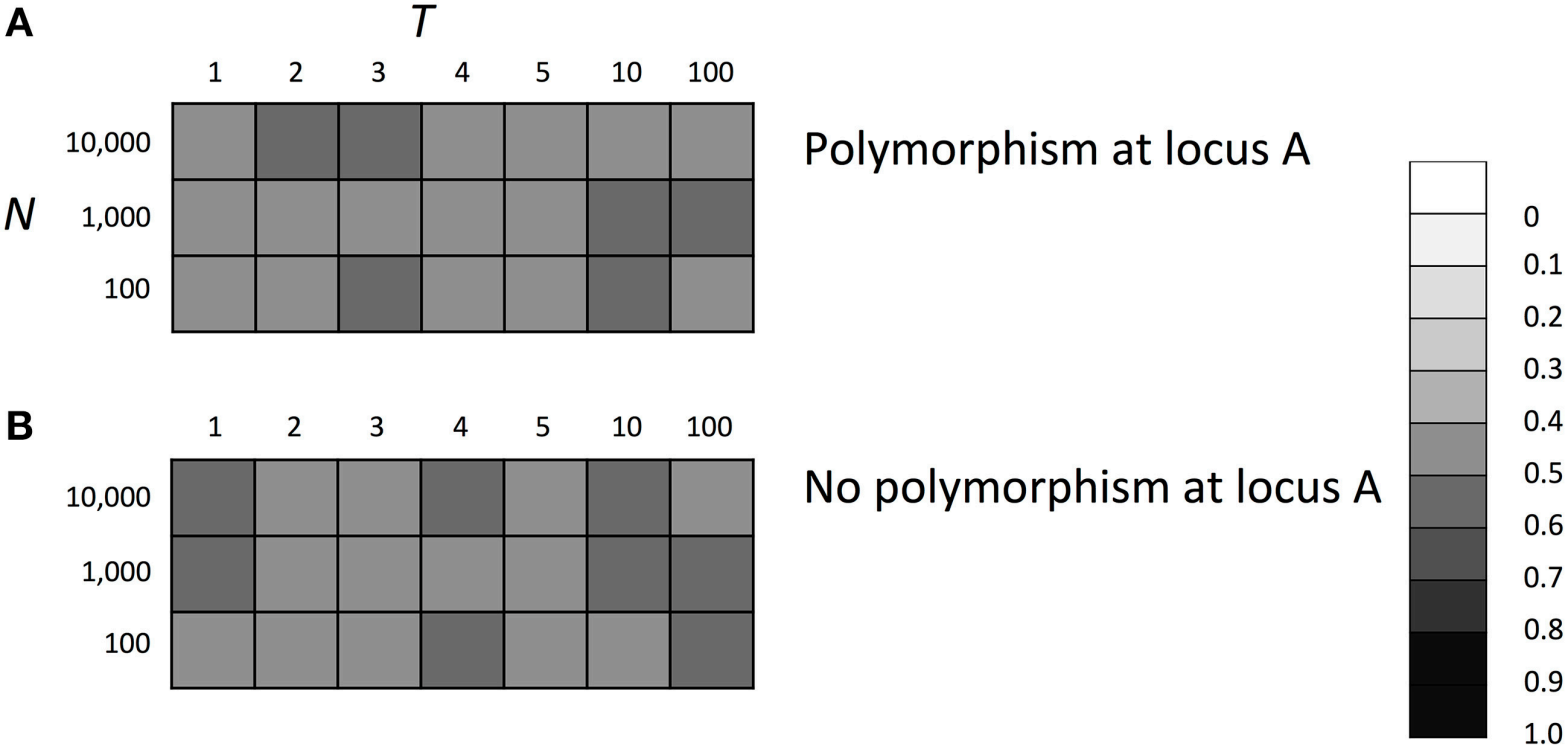
**The average frequency of SGE enhancing allele *B* under symmetric effect of SGE on fitness**. Viability of each genotype 1 + *q*, 1 − *h*_1_*S* + *q*, and 1 − *S* + *q* in environment 1, and 1 − *S* + *q*, 1 − *h*_2_*S* + *q*, and 1 + *q* in environment 2 for *AA, Aa*, and *aa*, respectively. The magnitude of SGE was regulated by locus *B* as σ_*BB*_ ≥ σ_*Bb*_ = *h*_b_^*^ σ_*BB*_ ≥ σ_*bb*_ = 0 (0 ≤ *h*_*b*_ ≤ 1). In this simulation, individuals were allowed to take fitness higher than 1, and the relative fitness to the highest fitness in the population was used as the mating success. Parameters *S* and σ_*BB*_ were set to 0.5, 0.1 respectively, and *h*_1_, *h*_2_, and *h*_*b*_ were set to 0.5 and the magnitude of SGE was regulated by locus *B* as σ_*BB*_ ≥ σ_*Bb*_ = *h*_b_^*^ σ_*BB*_ ≥ σ_*bb*_ = 0 (0 ≤ *h*_*b*_ ≤ 1). In panel **(A)**, Hardy-Weinberg equilibrium was assumed for locus A and B, and in panel **(B)**, allele *A* was assumed to be fixed at locus A. In each case, three population size *N* = 100, 1000, and 10,000 was considered. The simulation was replicated 1000 times for each parameter set and gray scale shows the average frequency of allele *B* after 1000 generations. In the simulation, constant population size, random mating, free recombination, and no mutation were assumed.

Thus far, we considered viability selection with the 0–1 boundary. This boundary can cause an asymmetric effect of SGE on fitness, which might influence the evolutionary fate of the SGE modifier gene. To evaluate the impact of this boundary on the results, we examined the evolutionary fate of a SGE modifier gene under the condition that the SGE effect on fitness was always symmetric. We considered the same selection scheme shown in Table 1 and removed the upper boundary 1 to allow individuals to take fitness higher than 1. The fitness of each individual was defined as the mating probability calculated by the relative value to the highest fitness in the population (fecundity selection or sexual selection). To make sure that fitness did not take a negative value, we considered *S* = 0.5 and σ_*BB*_ = 0.1, in which nearly 100% of individuals with disadvantageous genotypes could have absolute fitness >0. Thus, in this model and parameter set, the effect of SGE on fitness became symmetric and never changed the average fitness. For other parameters, we set *h*_1_ = *h*_2_ = *h*_*b*_ = 0.5 and considered three population size (*N* = 100, 1000, and 10,000). In Figure 6, we show the average frequency of allele *B* after 1000 generations. In contrast to the results under the viability selection, the SGE modifier gene evolved neutrally and took allele frequencies around 0.5 in changing environments when we assumed the symmetric effects of SGE on fitness. This result supports our suggestion that if there is a mechanism to make asymmetric effects of SGE on fitness and the average fitness is increased by SGE, enhanced SGE can be advantageous and evolve in the population as mentioned in Zhang et al. (2009).

## Discussion

In the present study, we tested a two-locus model in which one locus is under viability selection and the other one regulates the magnitude of SGE. Our results suggest that SGE acting on the gene under viability selection causes deleterious effects under the stable environment in our model. Our results also suggest that although enhanced SGE occasionally became advantageous in fluctuating environments, the enhanced SGE was disadvantageous in the majority of the parameter range. The major cause for the limited parameter range in which the enhanced SGE became advantageous in our model is our assumption of viability selection with a 0–1 boundary. Although it is beyond the scope of this study, the enhanced SGE might be advantageous in a wider parameter range when the viability of the adaptive basal genotype is lower than 1 and SGE can increase the fitness of individuals with the genotype. However, given that we assumed rather strong viability selection here, it would be inevitable for wild types to reach their adaptive optimal within a short evolutionary time. In other words, our results suggest that it is difficult for enhanced SGE to be advantageous in the population after fine-tuning adaptive evolution.

Compared to recently developed complex models (Draghi and Whitlock, 2015; Wolf et al., 2015), our model was designed much simpler in order to provide a foundation for future studies to evaluate more complicated case scenarios. Nevertheless, we believe that the main conclusions of this study are largely applicable to various real case scenarios, because many examples indicate that the expression level of a single or a few major genes can dramatically change the viability of individuals (Hou et al., 2009; Morran et al., 2011; Shi et al., 2012; Yang et al., 2012; Ravaux et al., 2013). Our results showed that in a stable environment, SGE acts as a noise, reduces the effective population size, and increases the fixation probability of slightly deleterious alleles, as predicted by Mineta et al. (2015) (Figure 2). In this case, SGE was always disadvantageous and the allele that enhances the magnitude of SGE could not increase its frequency in the population (Figure 3 and Supplementary Figure 2). Despite the disadvantageousness, SGE itself can be non-genetic and inevitably caused by both extrinsic and intrinsic molecular mechanisms (Elowitz et al., 2002; Raser and O'shea, 2005). Therefore, our result suggests that non-genetic SGE can significantly change the fixation probabiliy of slightly deleterious alleles and have an impact on the evolutionary processes of populations (See also Mineta et al., 2015).

In fluctuating environments, on the other hand, the gene which enhances the magnitude of SGE could be advantageous, especially if one allele was fixed at the locus directly contributing to the phenotypic trait (locus A; Figure 5). This result suggested that the enhanced SGE can be advantageous in fluctuating environments and was consistent with previous studies supporting the selective advantage of stochastic switching in fluctuating environments (Kussell and Leibler, 2005; Acar et al., 2008; Tsuru et al., 2011). However, with polymorphism at locus A, the conditions under which the enhanced SGE became advantageous were more tightly limited (Figure 4), which was most likely due to interactions between adaptive responses at loci A and B. Namely, extremely strong natural selection and frequent environment switch were necessary for the enhanced SGE to be advantageous. In this model, locus A can be adapted to the environment during *T* generations, which would have decreased the fitness of individuals with SGE after *T* generations. In other words, in a fluctuating environment, SGE can be advantageous just after the environmental change because at that time, most individual would have the genotype which is adaptive to the previous environment, and these individuals are disadvantageous without SGE. Still, polymorphism at locus A makes the adaptation to the new environment possible. Therefore, the advantage of SGE decreases as the frequency of the genotype adaptive to the new environment increases, and eventually SGE becomes disadvantageous. This is presumably why the enhanced SGE could not be advantageous when the interval between environmental switches (*T*) was long.

The advantageous effect of stochastic variation in phenotype has been suggested in previous studies (Bull, 1987; Gavrilets and Hastings, 1994; Kussell and Leibler, 2005; Acar et al., 2008; Salathe et al., 2009), and our results are consistent with these studies. However, our model incorporated the non-inherited stochastic fluctuation in phenotype caused by SGE, genetic control of SGE, and a 0–1 boundary of viability. As mentioned in the Introduction, SGE itself is non-genetic (Elowitz et al., 2002; Raser and O'shea, 2005), but the magnitude of SGE can be genetically regulated. Considering previous theoretical studies suggesting the importance of the production and degradation rate of protein and mRNA on the magnitude of SGE (Shahrezaei and Swain, 2008b; Li and Xie, 2011; Grima et al., 2012), genes that encode proteins functioning in these processes (e.g., RNA polymerase, nuclease or protein act as chaperon) might be SGE modifier gene candidates. Although Bull (1987) and Gavrilets and Hastings (1994) considered the genetic control of the fluctuation of phenotype and its non-inheritance, they assumed that the same locus regulates the basal phenotype and its fluctuation. Salathe et al. (2009) also considered the genetic control of the fluctuation of phenotype, but they assumed that the fluctuating phenotype is inherited to the next generations. Given that stochastic fluctuation in phenotype would not be inherited unless it occurred in the germ cell, our assumption of non-inherited SGE would be realistic. In addition, there are several reports showing examples that the expression level of a single or a few major genes, which relate to stress or disease tolerance, can dramatically change individuals' viability (Hou et al., 2009; Morran et al., 2011; Shi et al., 2012; Yang et al., 2012; Ravaux et al., 2013). Our results can be applicable to these examples in principle although a more complex model is required to explain the detailed empirical data in future studies.

Our result also suggested the importance of the degree of dominance at the locus affected by natural selection for the evolutionary fate of the SGE modifier gene (Supplementary Figure 6). The degree of dominance at these loci regulates the fitness of heterozygotes and therefore, affects the advantage of SGE as mentioned above. The maintenance of the polymorphism in heterozygotes is a feature of diploid organisms and was not considered in the previous studies (Kussell and Leibler, 2005; Acar et al., 2008). If the deleterious allele in one environment is dominant, natural selection can efficiently decrease the frequency of this allele and therefore, the advantage of the enhanced SGE just after the environmental change becomes large. On the other hand, if the deleterious allele is recessive, a population can maintain this allele with relatively high frequency and decrease the advantage of SGE. In contrast to the locus under natural selection (locus A), the degree of dominance at the SGE modifier gene (locus B) showed only a small effect on the parameter range in which enhanced SGE becomes advantageous (Supplementary Figure 7). This may be because of the initial condition of Hardy-Weinberg equilibrium at locus B, under which individuals can easily make homozygote of the advantageous allele at this locus. If the initial frequency of the advantageous allele is low and almost all of advantageous alleles are heterozygous, degree of dominance would become important. Although we did not consider other initial condition, if the initial frequency of allele *B* is very low, the effect of *h*_*b*_ might become larger. In addition, we found that small *h*_*b*_ prevents the enhanced SGE from being removed from the population in wide parameter ranges. Even if the enhanced SGE was not advantageous, the maintenance of allele *B* would increase the probability of evolution of enhanced SGE by genetic drift in this case.

Finally, we will briefly discuss one interesting feature of SGE we found. As discussed above, in most cases, SGE acts as a noise which decreases individual's fitness. However, SGE was found to be capable of increasing heterozygosity at the selectively neutral locus when there was strong selection under a fluctuating environment even if the enhanced SGE itself was disadvantageous (Supplementary Figure 8). This means that SGE can contribute to the maintenance of genetic diversity within the population, supporting that SGE was indeed not just a noise in the models of the fluctuating environment. Given the fact that environments and their effects on living organisms do fluctuate in many ways in nature (Rees et al., 2004), the potential contribution of SGE to the level of genetic diversity and effective population size may also be of evolutionary importance.

## Conclusions

Although it is difficult for enhanced SGE to be advantageous, SGE itself affects the genetic structure of the population; increasing fixation probability of slightly deleterious mutations in stable environments and increasing heterozygosity in fluctuating environments. The enhanced SGE could be advantageous only in very tight, and maybe unrealistic conditions for diploid organisms. However, like the effect of the degree of dominance, other genetic models might affect the evolutionary fate of the SGE modifier gene. Note here that our simulations largely neglected genomic heterogeneity and complex genetic architecture with which SGE in one gene can be indirectly associated with phenotypic variance. It is also noteworthy that linkage disequilibrium between a SGE modifier gene and its target gene might alter the fate of SGE evolution. As discussed in Mineta et al. (2015), there are several sources of SGE. Some papers mentioned that the competition between transcription factors and nucleosomes cause SGE (Tirosh and Barkai, 2008; Choi and Kim, 2009; Macneil and Walhout, 2011). Others mentioned that cluster of essential genes around open chromatin leads to more robust expression levels (Batada and Hurst, 2007; Field et al., 2008). These cases including more realistic number of loci relating to the phenotype should be examined in the future studies both theoretically and experimentally.

## Funding

This work was supported by the Swiss National Science Foundation Grant to HA (No. 31003A_125213).

### Conflict of interest statement

The authors declare that the research was conducted in the absence of any commercial or financial relationships that could be construed as a potential conflict of interest.
