# Supplementary material for: An Individual-Based Diploid Model Predicts Limited Conditions Under Which Stochastic Gene Expression Becomes Advantageous
Source: Front Genet. 2015 Nov 24;6:336. doi: 10.3389/fgene.2015.00336 (PMC4656826; doi:10.3389/fgene.2015.00336)
Supplement: Supplementary file 1 [file DataSheet1.DOCX]

***Supplementary Material***

**An individual-based diploid model predicts limited condition in which stochastic gene expression becomes advantageous under fluctuating environment**

**Tomotaka Matsumoto, Katsuhiko Mineta, Naoki Osada, Hitoshi Araki^*^**

**Correspondence:** Hitoshi Araki, Ph.D: [arakih@res.agr.hokudai.ac.jp](mailto:arakih@res.agr.hokudai.ac.jp)

Supplementary Figure 1. The average and standard deviation of allele frequency of allele *A* under stable environment 1. The model was the same to that used in Fig. 2. We considered three *S* values, (A) *S* = 0, (B) *S* = 0.5 and (C) *S* = 0.9, and four *σ*_BB_ values, 0.01, 0.1, 0.5 and 1 represented as different line style. The simulation was replicated 1,000 times for each parameter set.


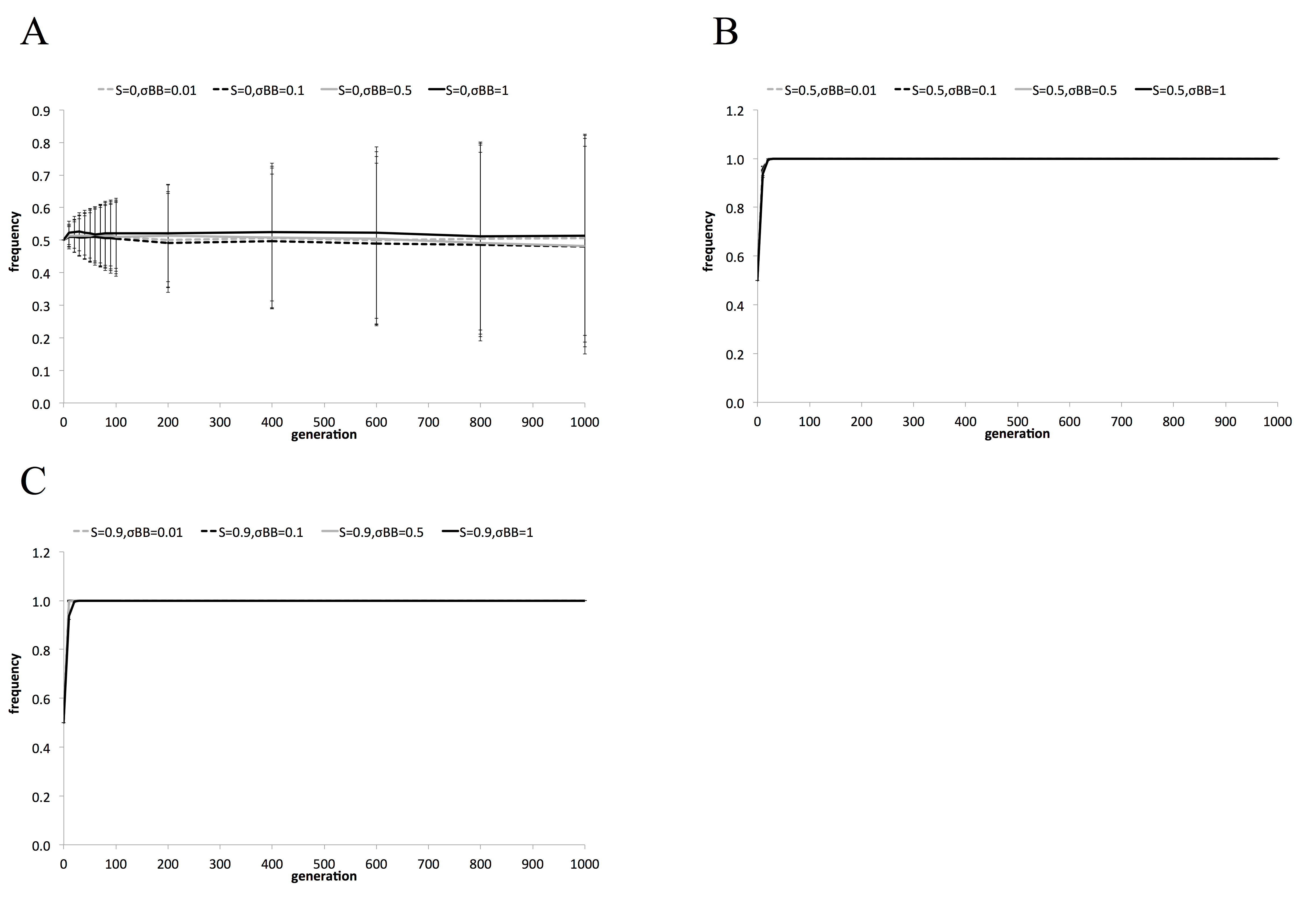


**Supplementary Figure 2. The average and standard deviation of allele frequency of allele *B* under stable environment 1.** The model was the same to that used in Fig. 2. We considered three *S* values, (A) *S* = 0, (B) *S* = 0.5 and (C) *S* = 0.9, and four *σ*_BB_ values, 0.01, 0.1, 0.5 and 1 represented as different line style. The simulation was replicated 1,000 times for each parameter set.


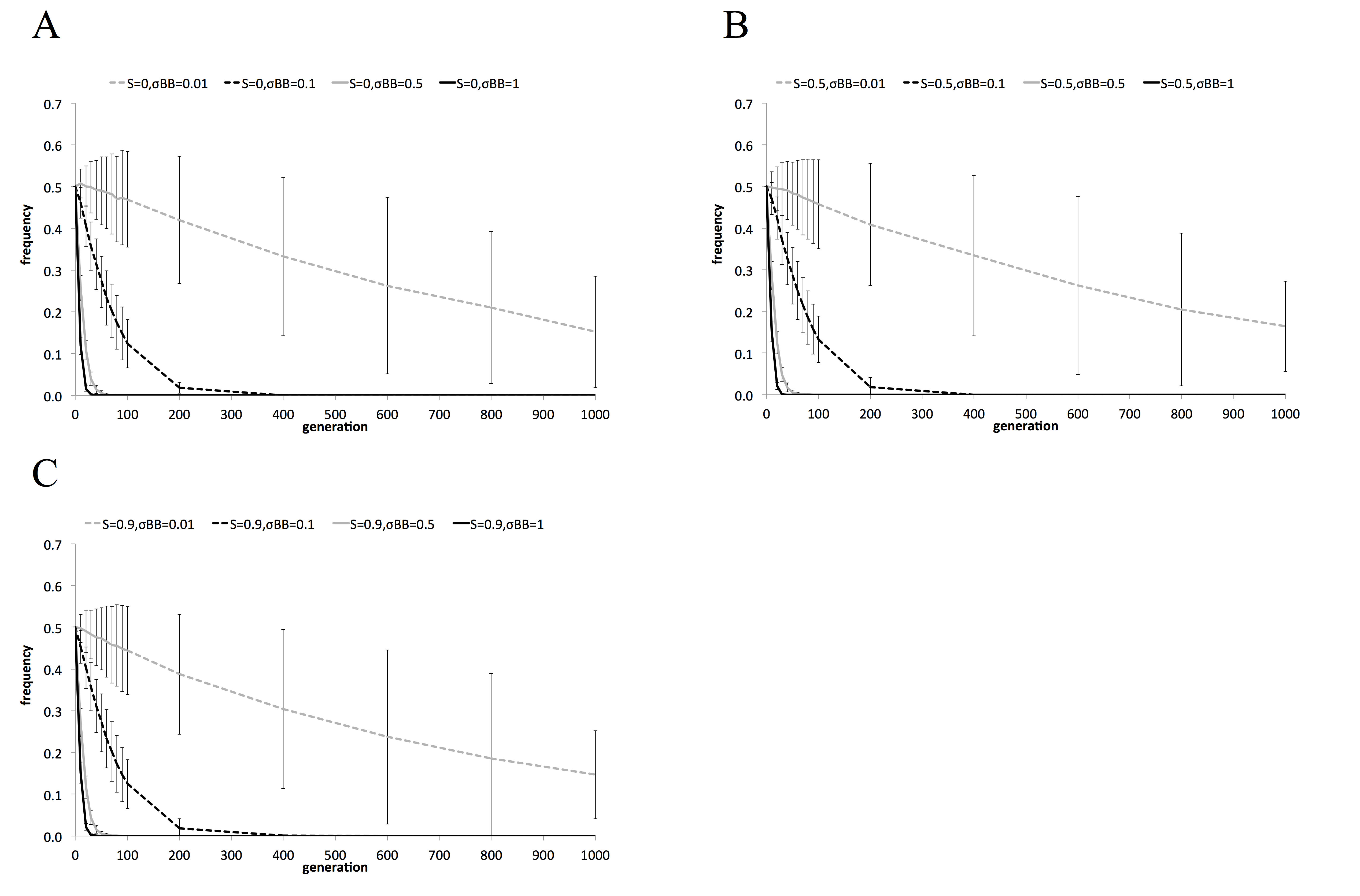


**Supplementary Figure 3. The average and standard deviation of allele frequency of allele *A* under fluctuating environments.** The model was the same to that used in Fig. 3. We considered four combinations of parameter *S* and *T*, (A) *S* = 0.8, *T* = 10, (B) *S* = 0.8, *T* = 1, (C) *S* = 0.9, *T* = 10 and (D) *S* = 0.9, *T* = 1, and four *σ*_BB_ values, 0.01, 0.1, 0.5 and 1 represented as different line style. The simulation was replicated 1,000 times for each parameter set.


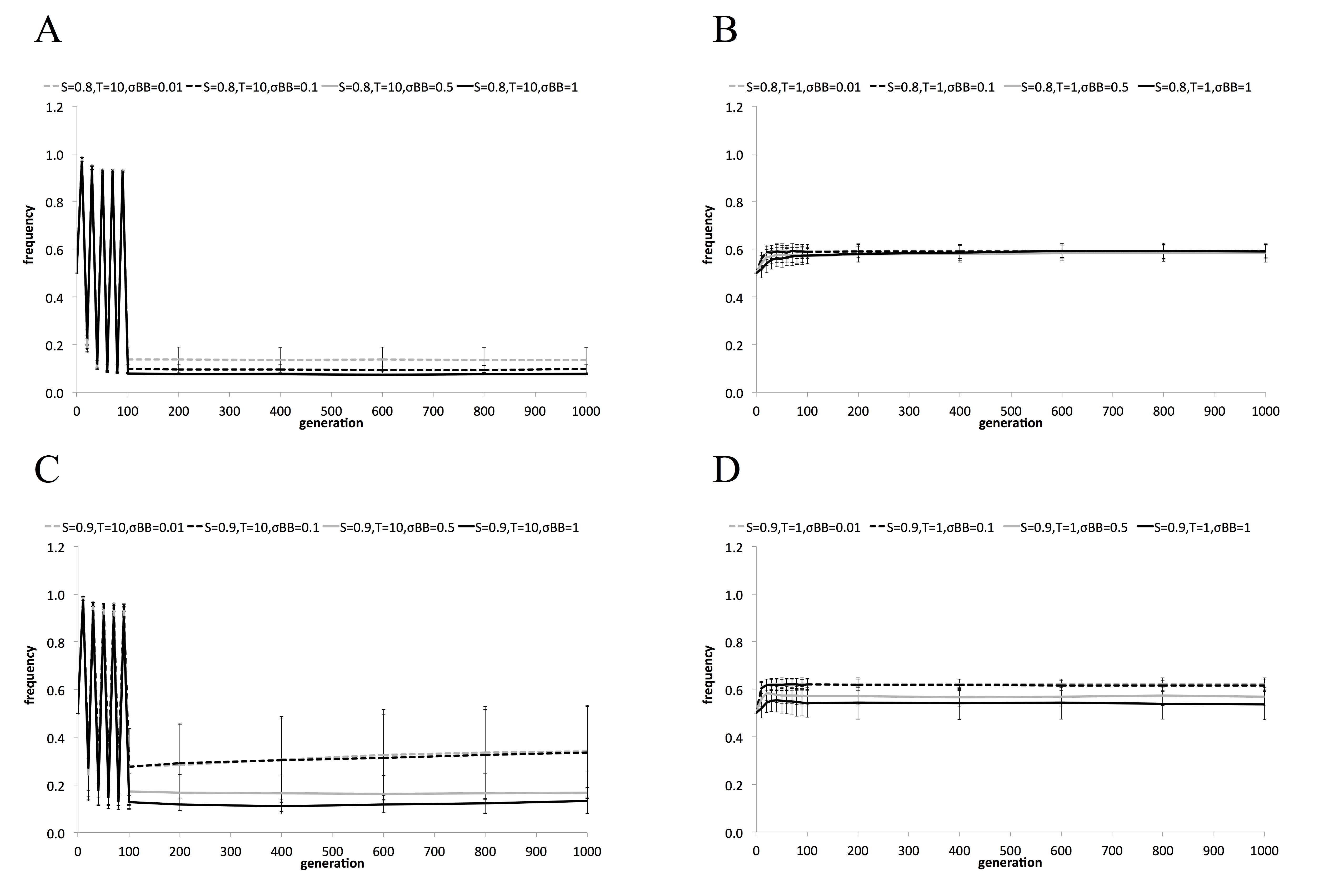


**Supplementary Figure 4. The average and standard deviation of allele frequency of allele *B* under fluctuating environments.** The model was the same to that used in Fig. 3. We considered four combinations of parameter *S* and *T*, (A) *S* = 0.8, *T* = 10, (B) *S* = 0.8, *T* = 1, (C) *S* = 0.9, *T* = 10 and (D) *S* = 0.9, *T* = 1, and four *σ*_BB_ values, 0.01, 0.1, 0.5 and 1 represented as different line style. The simulation was replicated 1,000 times for each parameter set.


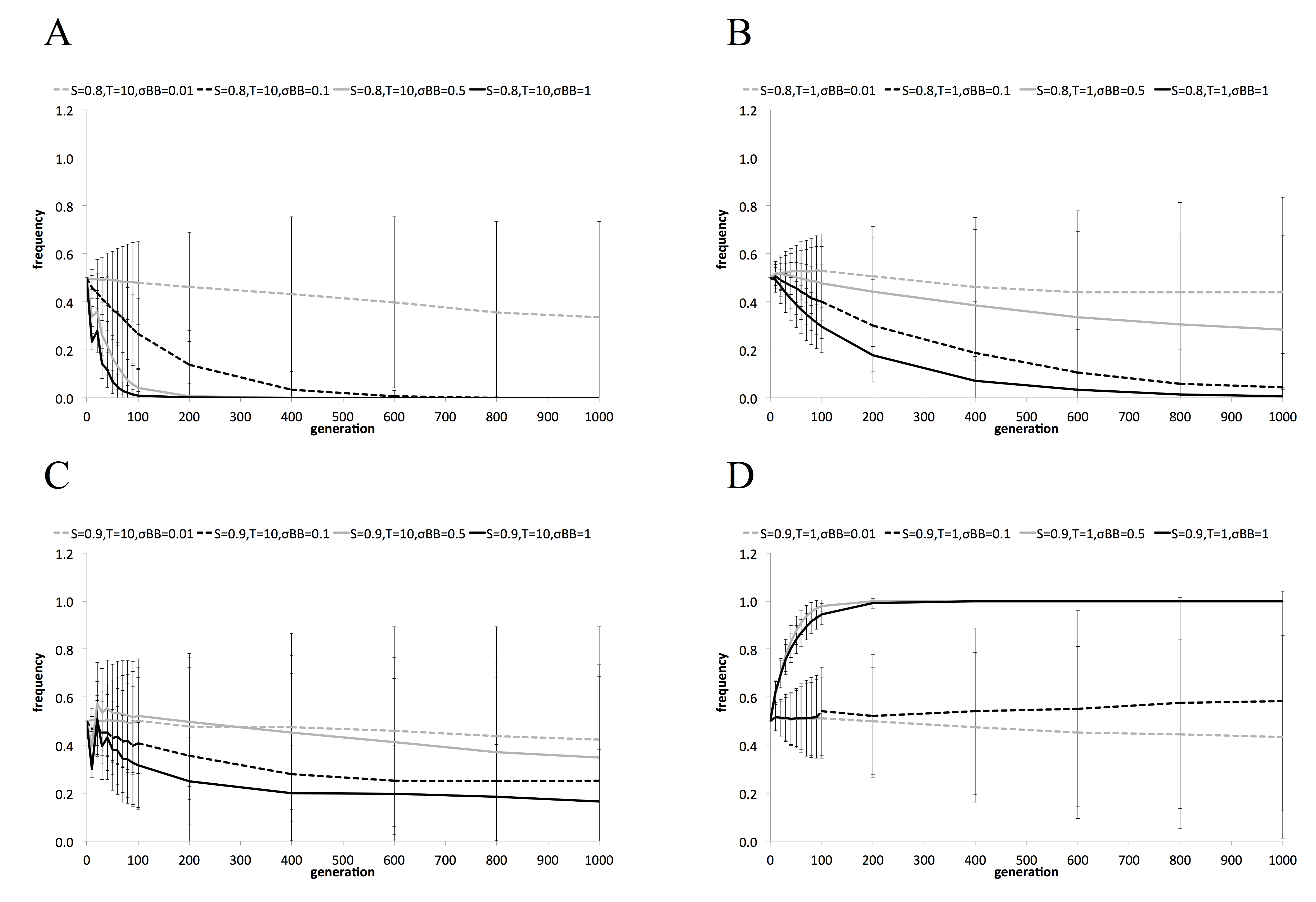


**Supplementary Figure 5. The average and standard deviation of allele frequency of allele *B* under fluctuating environments assuming fixed allele *A*.** The model and parameter values were same to that used in Fig. 4. We considered two combinations of parameter *S* and *T*, (A) *S* = 0.8, *T* = 10, (B) *S* = 0.9, *T* = 10, and four *σ*_BB_ values, 0.01, 0.1, 0.5 and 1 represented as different line style. The simulation was replicated 1,000 times for each parameter set.


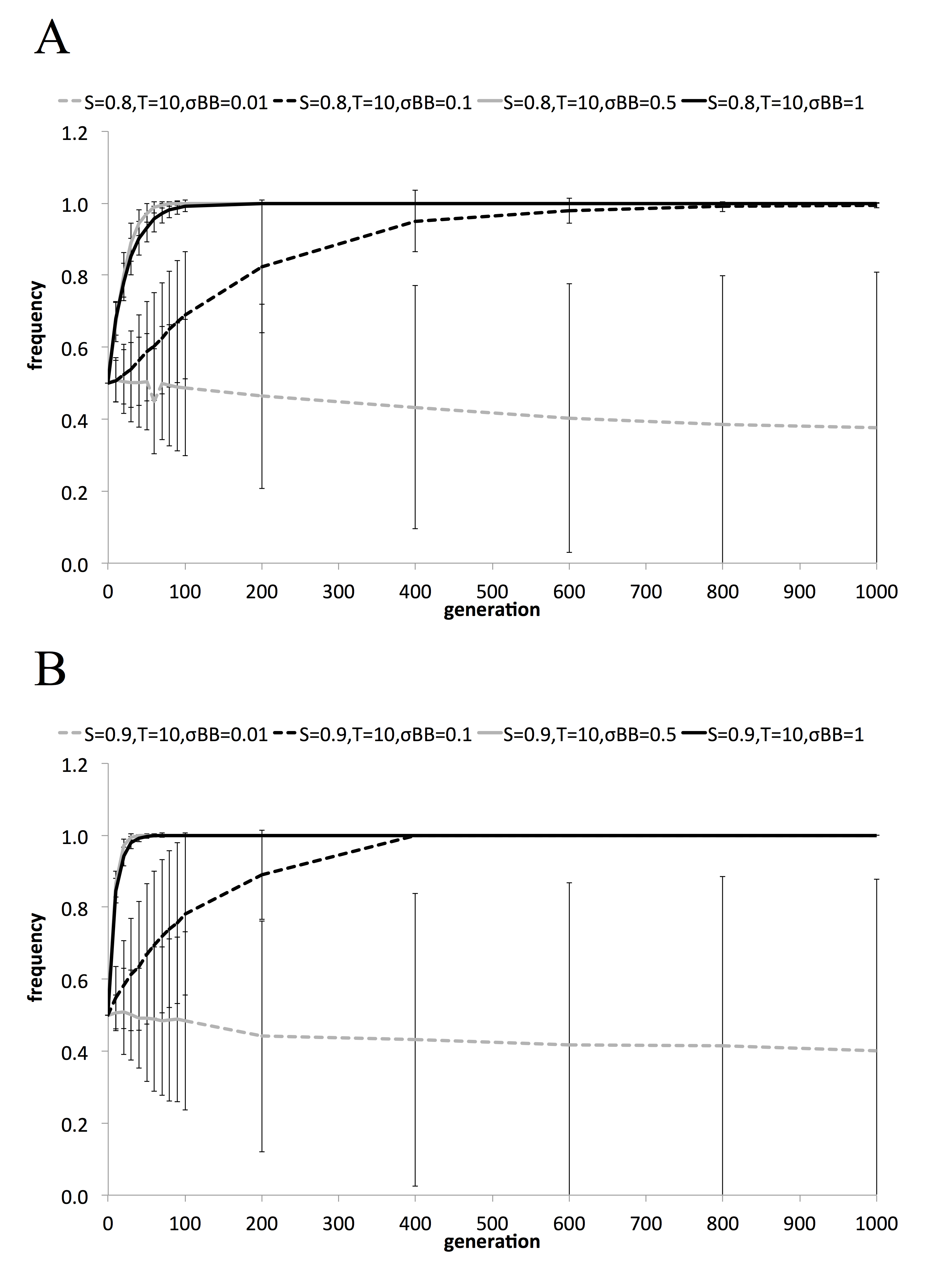


**Supplementary Figure 6. The average frequency of SGE enhancing allele *B* under fluctuating environments assuming different degree of dominance of locus A.** The model was the same to that used in Fig. 3. We considered *σ*_BB_ = 1.0 and four cases of the degree of dominance, (A) *h*_1_ = 0, *h*_2_ = *h*_b_ = 0.5, (B) *h*_2_ = 0, *h*_1_ = *h*_b_ = 0.5, (C) *h*_1_ = 1, *h*_2_ = *h*_b_ = 0.5 and (D) *h*_2_ = 1, *h*_1_ = *h*_b_ = 0.5. The simulation was replicated 1,000 times for each parameter set and gray scale shows the average frequency of allele *B* after 1,000 generations.


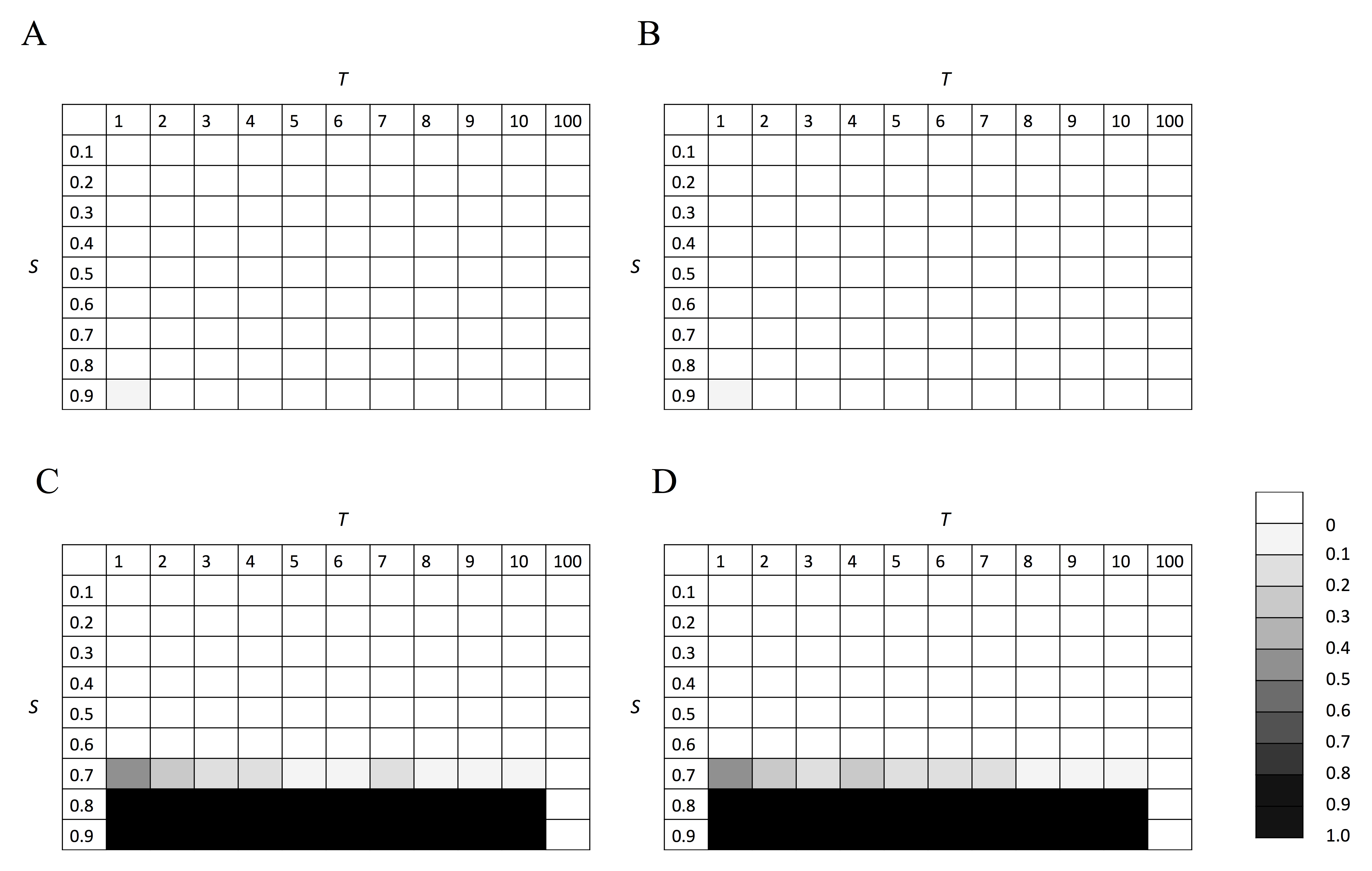


**Supplementary Figure 7. The average frequency of SGE enhancing allele *B* under fluctuating environments assuming different degree of dominance of locus B.** The model was the same to that used in Fig. 3. We considered *σ*_BB_ = 1.0 and two cases of the degree of dominance, (A) *h*_b_ = 0, *h*_1_ = *h*_1_ = 0.5 and (B) *h*_b_ = 1, *h*_1_ = *h*_2_ = 0.5. The simulation was replicated 1,000 times for each parameter set and gray scale shows the average frequency of allele *B* after 1,000 generations.


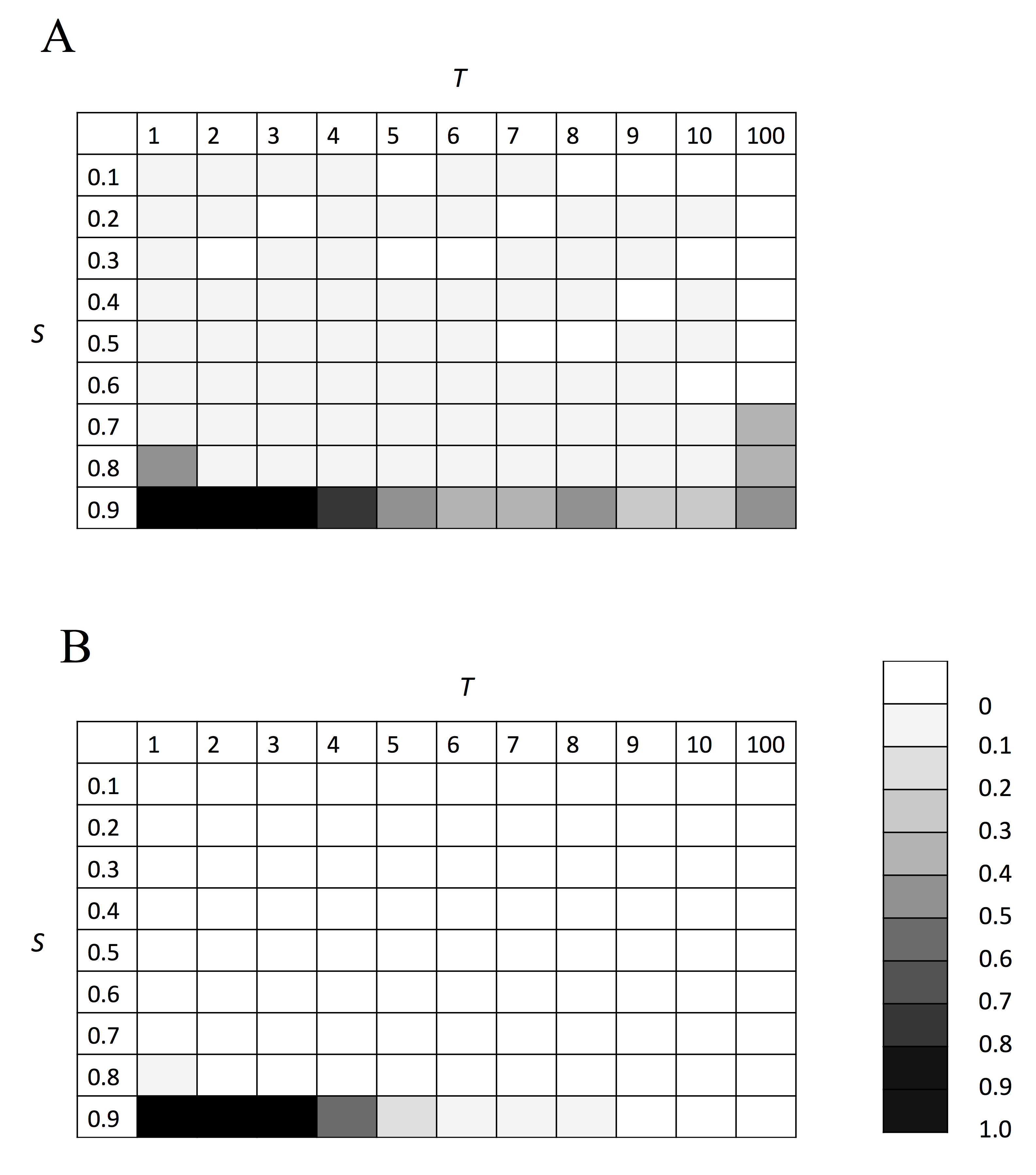


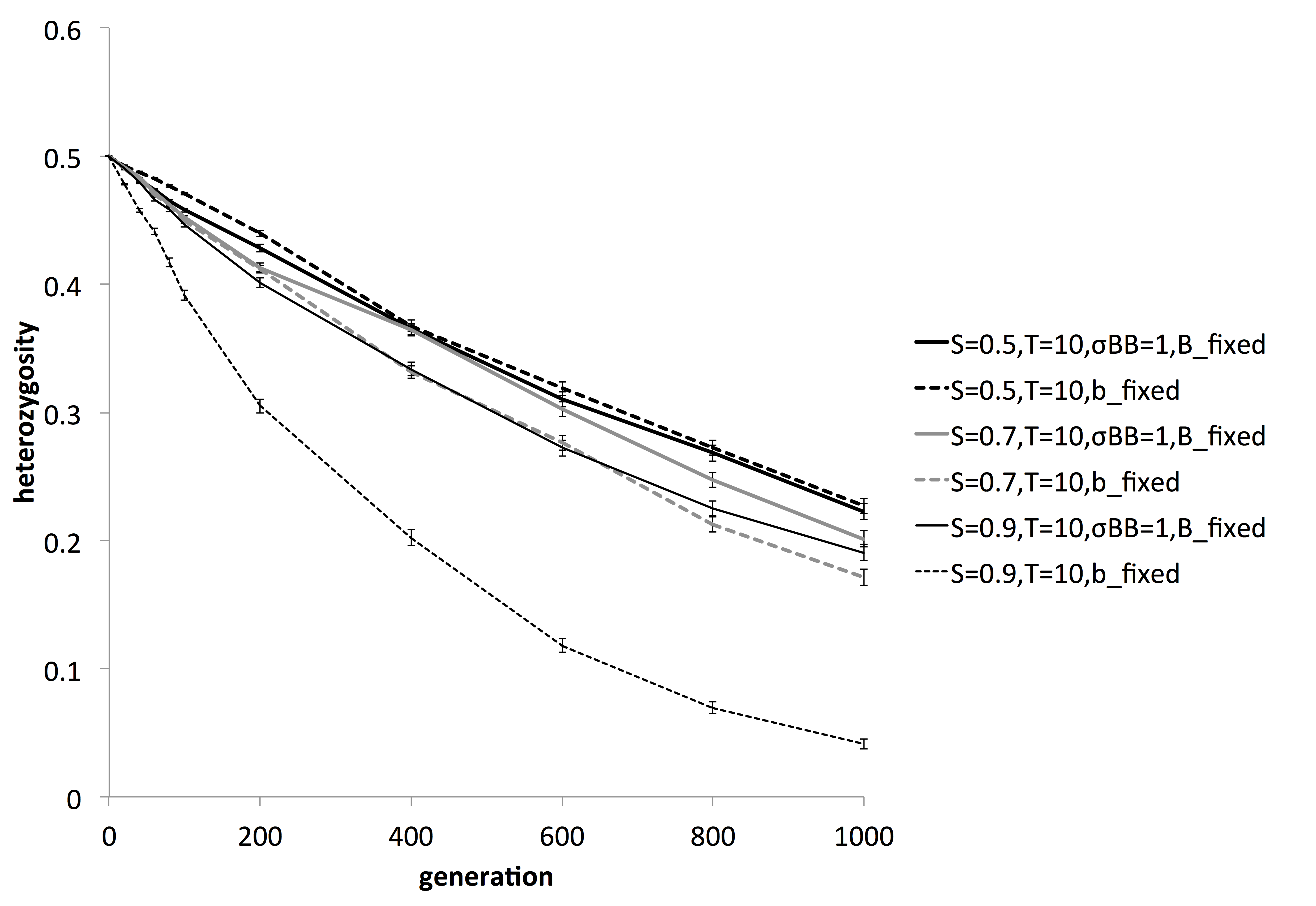
**Supplementary Figure 8. Average and standard error of heterozygosity of selectively neutral locus *C* under fluctuating environment.** To examine heterozygosity of selectively neutral locus, we added one locus C with two alleles *C* and *c* in our model. Locus C was assumed to be selectively neutral. Viability of each genotype 1 + *q*, 1 – *h*_1_*S* + *q*, and 1 – *S* + *q* in environment 1, and 1 – *S* + *q*, 1 – *h*_2_*S* + *q*, and 1 + *q* in environment 2 for *AA*, *Aa*, and *aa*, respectively. Two environments switched every *T* generation. The magnitude of SGE was regulated by locus B as *σ_BB_* ≥ *σ_bb_* = 0, and thus, *B* fixed case was SGE case and *b* fixe case was no-SGE case. We considered three combinations of parameter *S* and *T*, (1) *S* = 0.5, *T* = 10, (2) *S* = 0.7, *T* = 10, (3) *S* = 0.9, *T* = 10 represented as different line style. As the initial condition, Hardy-Weinberg equilibrium was assumed for locus A and *C.* We also set *N* = 1,000, *h*_1_ = *h*_2_ = 0.5. The simulation was replicated 1,000 times for each parameter set assuming constant population size, random mating, free recombination and no mutation.
