# Supplementary figures and images for: An Individual-Based Diploid Model Predicts Limited Conditions Under Which Stochastic Gene Expression Becomes Advantageous
Source: Front Genet. 2015 Nov 24;6:336. doi: 10.3389/fgene.2015.00336 (PMC4656826; doi:10.3389/fgene.2015.00336)

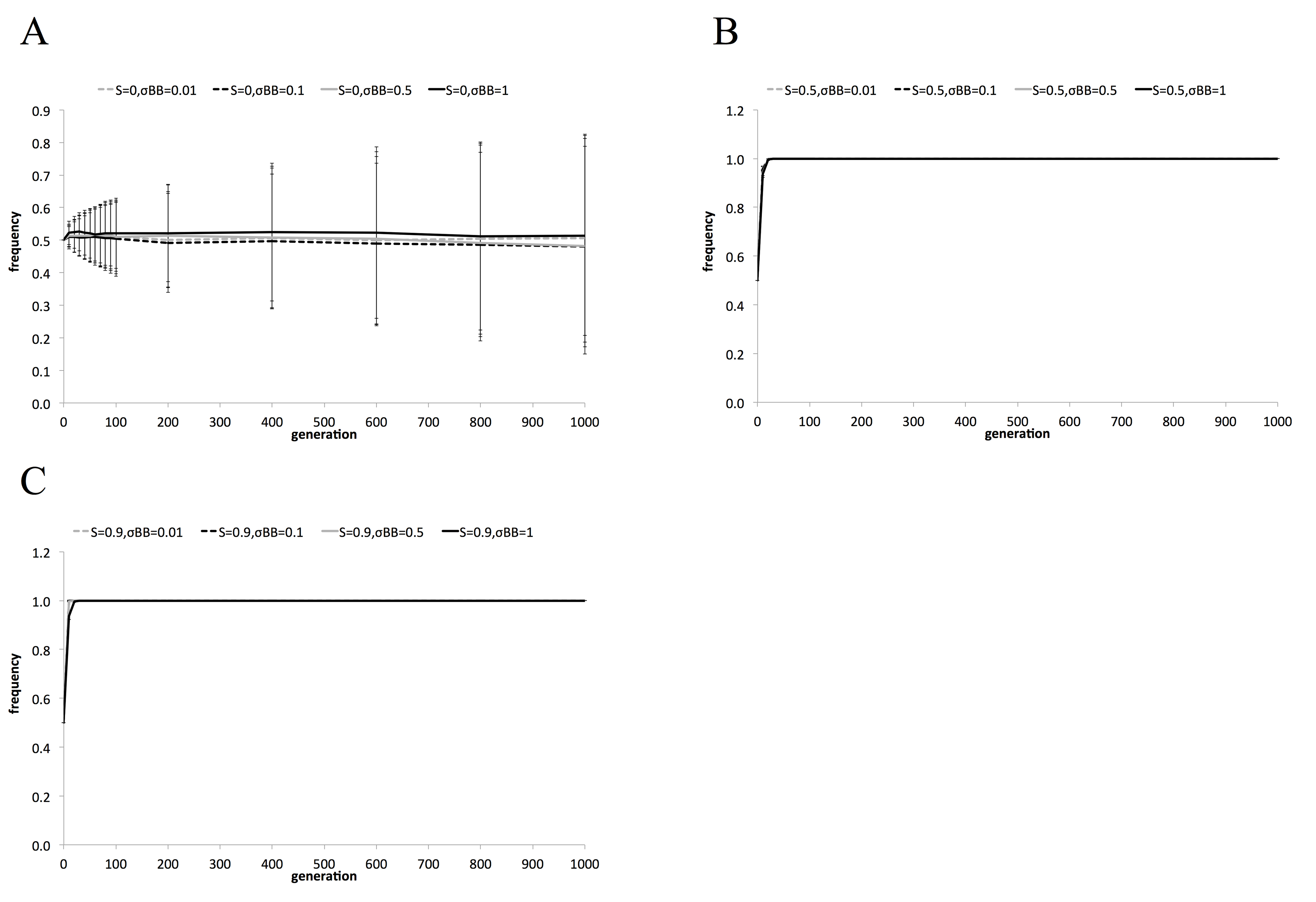

Supplement: Supplementary file 2 [file Image1.TIFF]

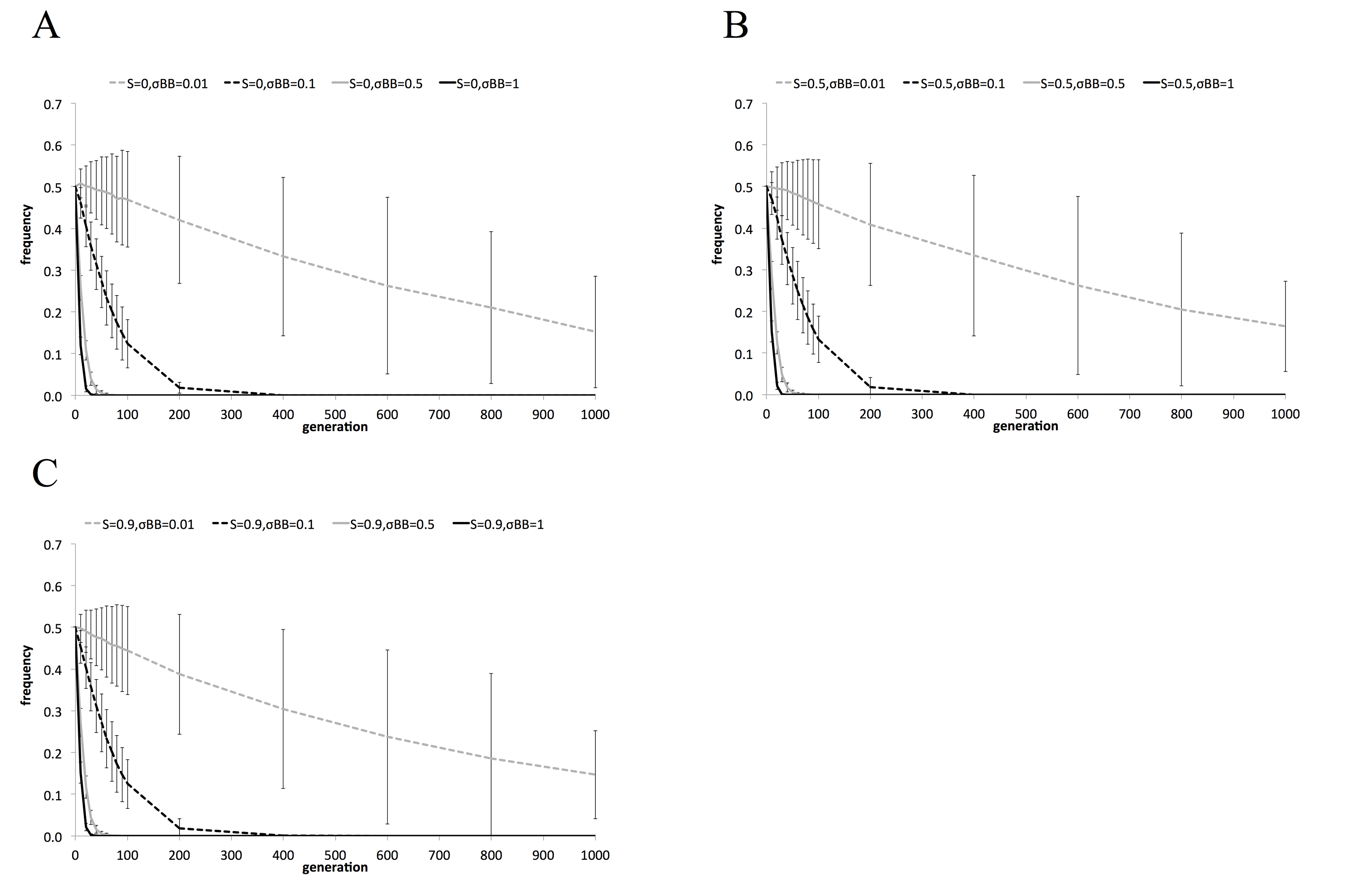

Supplement: Supplementary file 3 [file Image2.TIFF]

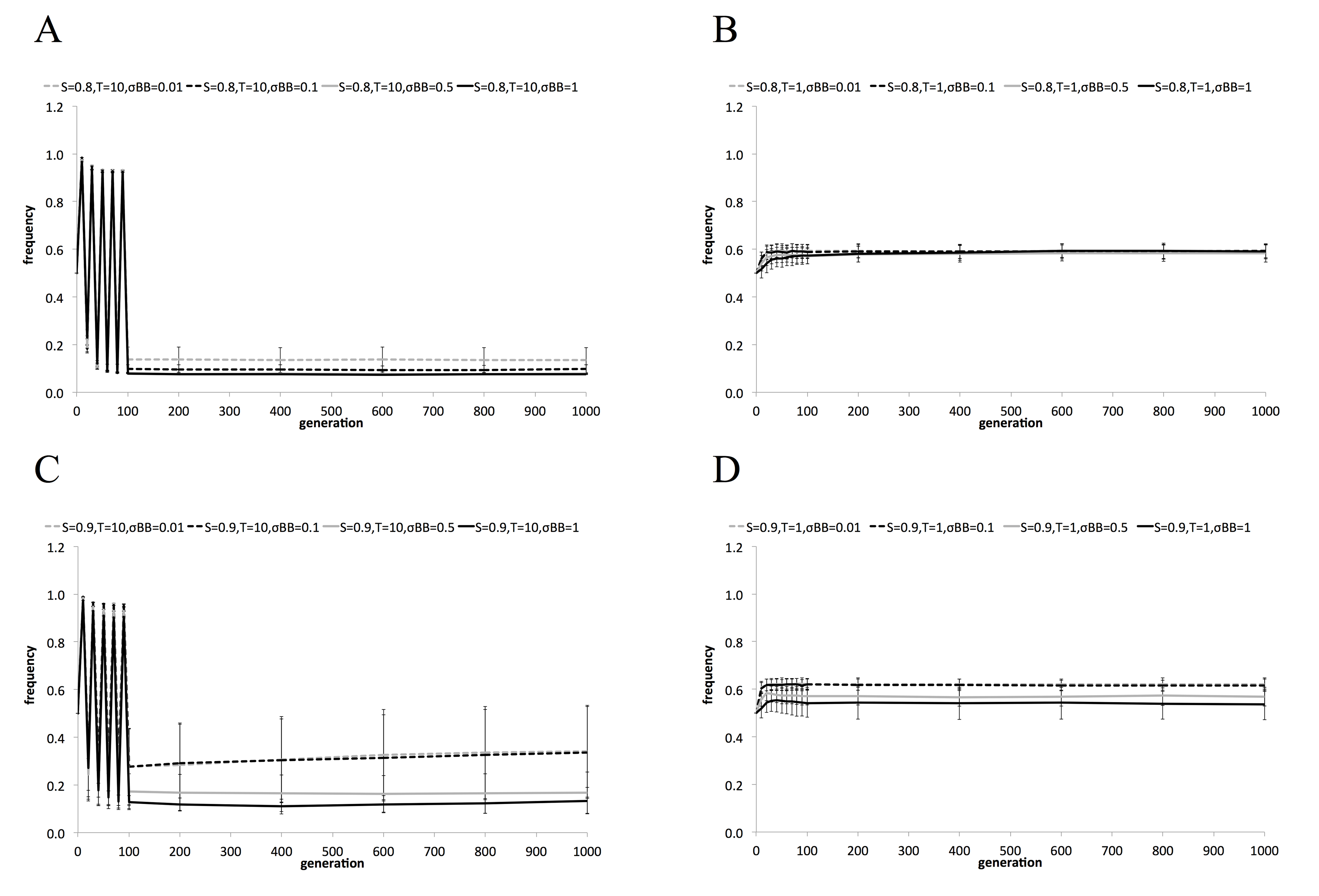

Supplement: Supplementary file 4 [file Image3.TIFF]

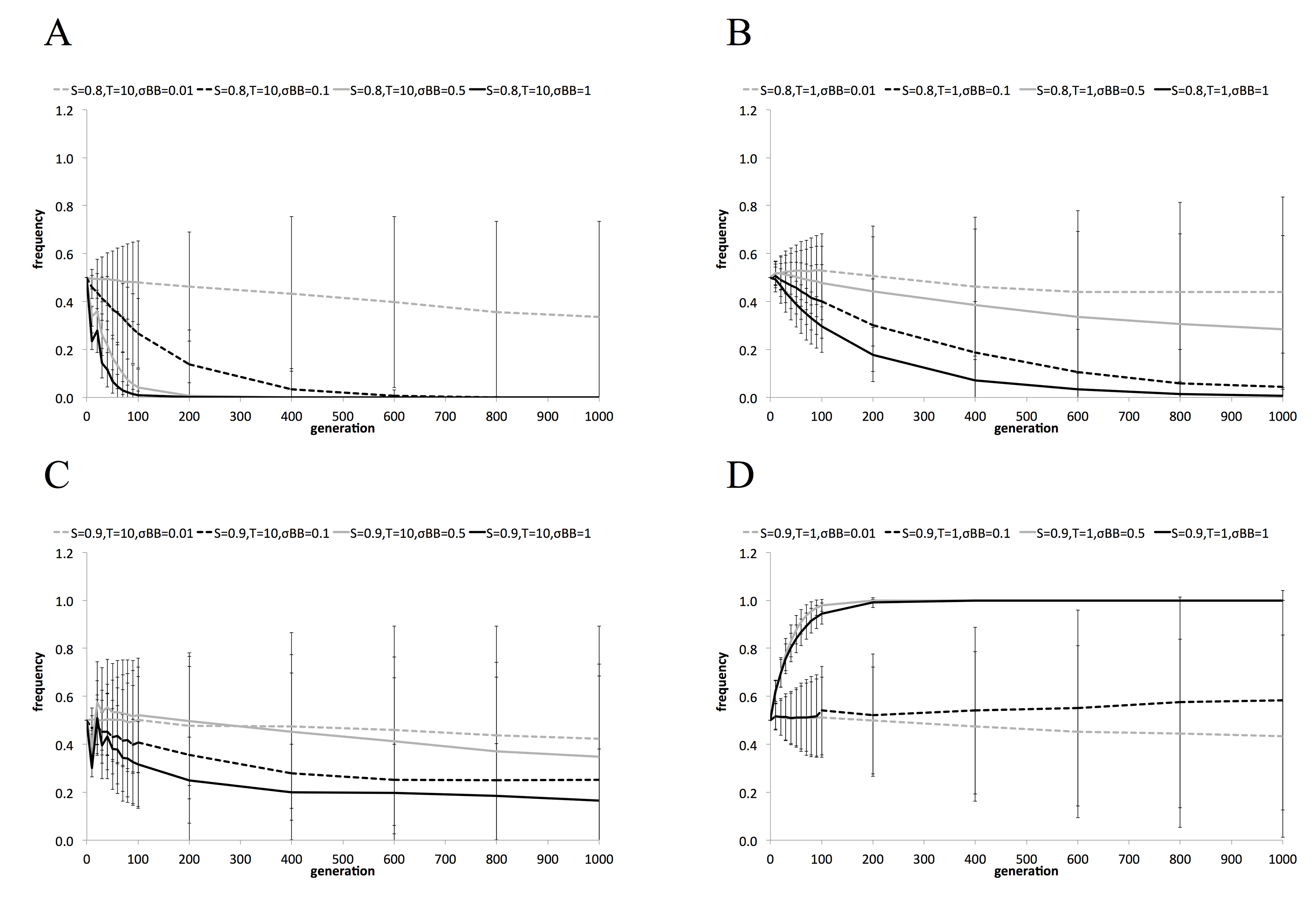

Supplement: Supplementary file 5 [file Image4.TIFF]

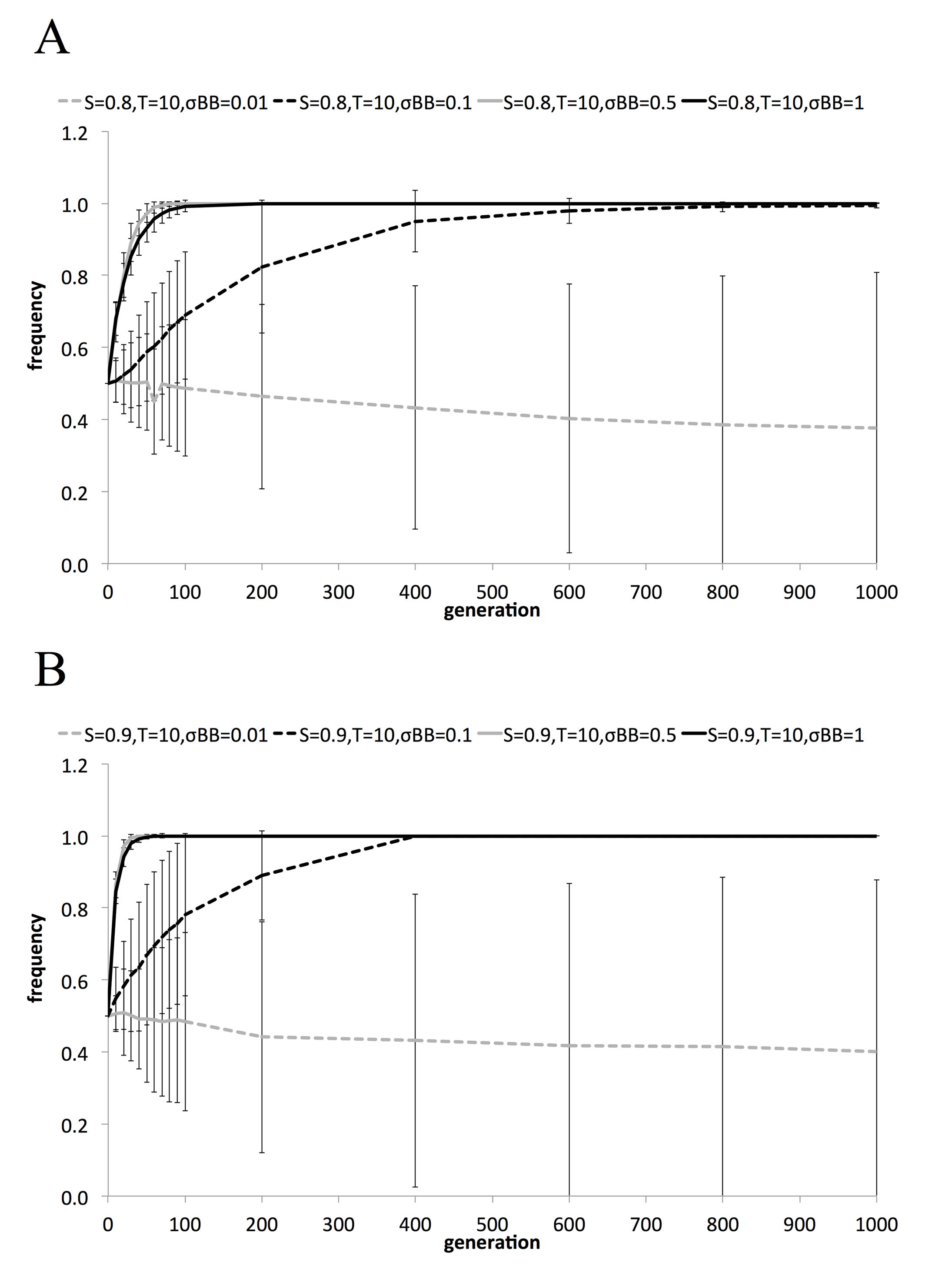

Supplement: Supplementary file 6 [file Image5.TIFF]

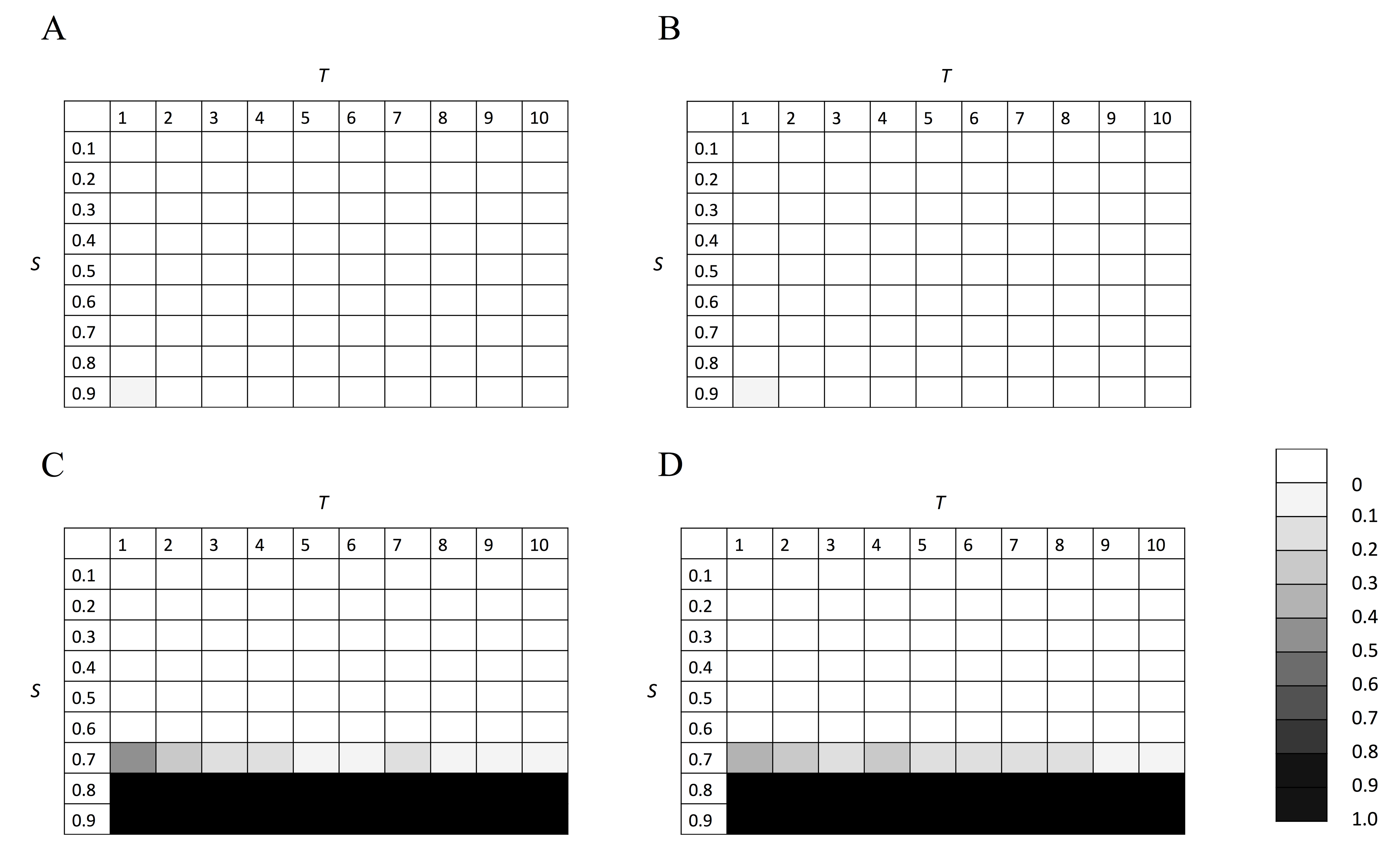

Supplement: Supplementary file 7 [file Image6.TIFF]

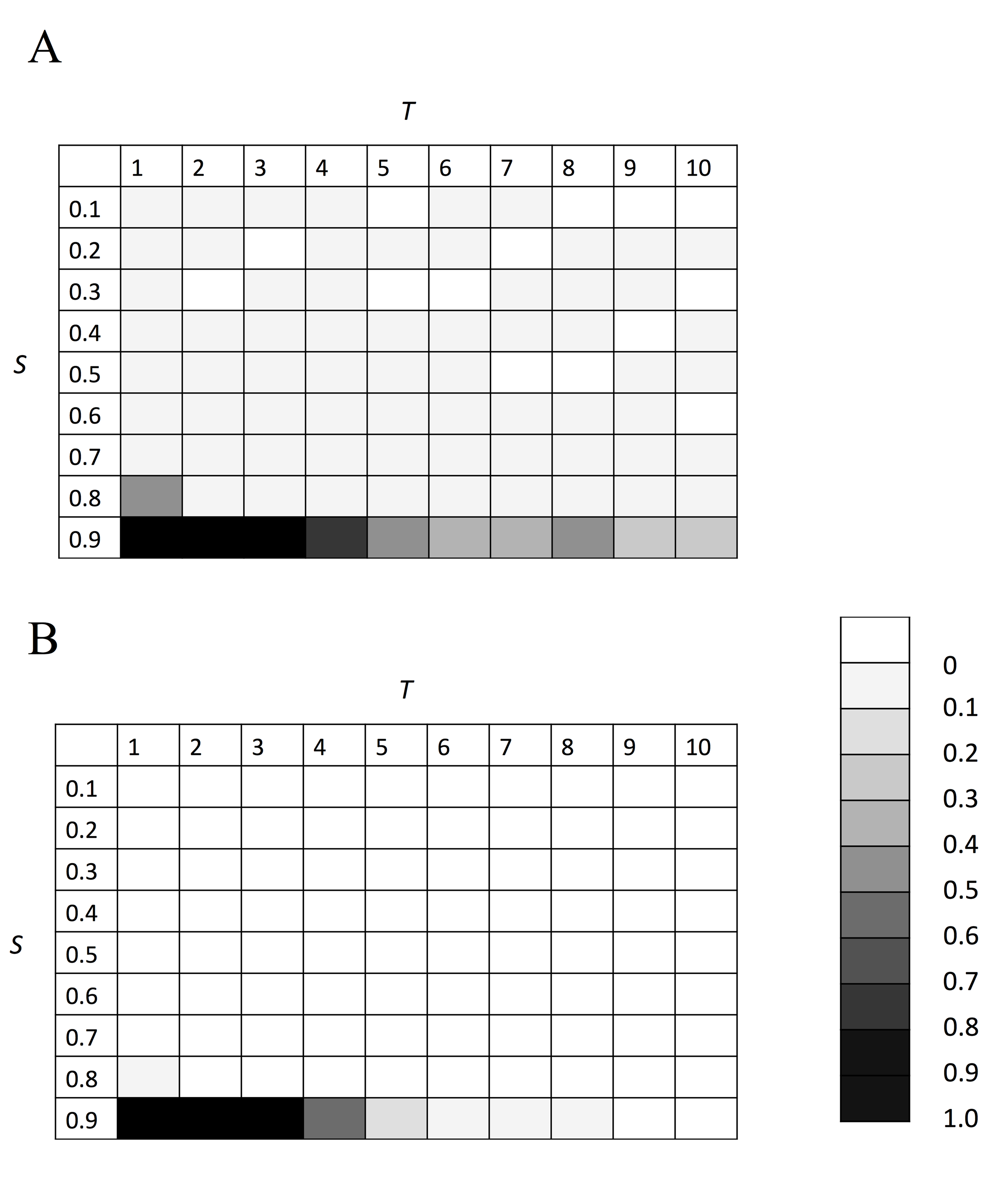

Supplement: Supplementary file 8 [file Image7.TIFF]

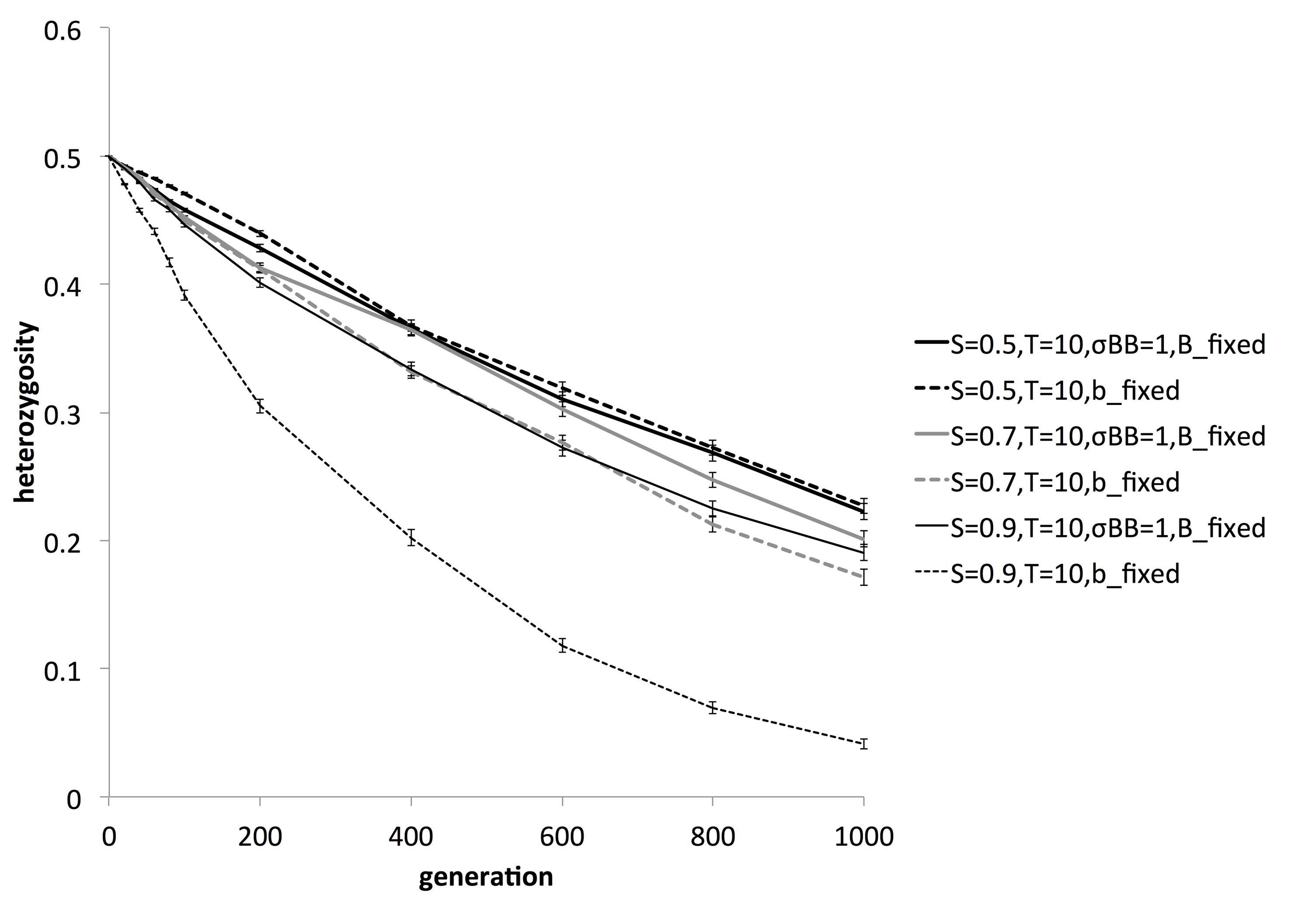

Supplement: Supplementary file 9 [file Image8.TIFF]
